# Genomic studies controvert the existence of the CUX1 p75 isoform

**DOI:** 10.1038/s41598-021-03930-4

**Published:** 2022-01-07

**Authors:** Manisha Krishnan, Madhavi D. Senagolage, Jeremy T. Baeten, Donald J. Wolfgeher, Saira Khan, Stephen J. Kron, Megan E. McNerney

**Affiliations:** 1 Committee on Cancer Biology, The University of Chicago, Chicago, IL USA; 2 Department of Molecular Genetics and Cell Biology, The University of Chicago, Chicago, IL USA; 3 Department of Pathology, The University of Chicago, Chicago, IL USA; 4 Department of Pediatrics, The University of Chicago, Chicago, IL USA; 5 The University of Chicago Medicine Comprehensive Cancer Center, University of Chicago, Chicago, IL USA

**Keywords:** Cancer, Genetics

## Abstract

*CUX1*, encoding a homeodomain-containing transcription factor, is recurrently deleted or mutated in multiple tumor types. In myeloid neoplasms, *CUX1* deletion or mutation carries a poor prognosis. We have previously established that CUX1 functions as a tumor suppressor in hematopoietic cells across multiple organisms. Others, however, have described oncogenic functions of CUX1 in solid tumors, often attributed to truncated CUX1 isoforms, p75 and p110, generated by an alternative transcriptional start site or post-translational cleavage, respectively. Given the clinical relevance, it is imperative to clarify these discrepant activities. Herein, we sought to determine the CUX1 isoforms expressed in hematopoietic cells and find that they express the full-length p200 isoform. Through the course of this analysis, we found no evidence of the p75 alternative transcript in any cell type examined. Using an array of orthogonal approaches, including biochemistry, proteomics, CRISPR/Cas9 genomic editing, and analysis of functional genomics datasets across a spectrum of normal and malignant tissue types, we found no data to support the existence of the CUX1 p75 isoform as previously described. Based on these results, prior studies of p75 require reevaluation, including the interpretation of oncogenic roles attributed to CUX1.

## Introduction

Protein isoforms and splice variants often have important and distinct biological functions. Since protein isoforms, by definition, have considerable sequence homology and may be expressed at different levels within the cell, it can be challenging to accurately differentiate between and functionally characterize such isoforms^1^. One such protein reported to have multiple isoforms is CUT-like homeobox 1 (CUX1), a HOX-family transcription factor with critical roles in development and tumorigenesis. In vertebrates, the *CUX1* locus contains two distinct genes that partially share exons: *CUX1*, which encodes a transcription factor localized to the nucleus, and *CASP*, which encodes a golgi-associated transmembrane protein involved in retrograde transport^2–4^. Altered levels and mutations of the *CUX1* transcription factor have been implicated in cancer across several tumor types and species^5,6^. *CASP*, on the other hand, has not been implicated in human disease^7,8^. The RefSeq database documents seven mRNA isoforms for the human *CUX1* locus; five of these are *CASP* transcripts and two are *CUX1* (Fig. S1). Due to its relevance to human health, we focus our attention herein on *CUX1*. For the sake of simplicity, our subsequent references to the *CUX1* gene or mRNA allude to those isoforms that encode CUX1, unless stated otherwise.

CUX1 is highly conserved, ubiquitously expressed, and essential for survival in mice and *Drosophila*^9^. CUX1 controls many cellular processes including determination of cell identity, cell cycle progression, cell–cell communication, and cell motility^9^. In cancer, however, there are conflicting reports of *CUX1* acting alternately as an oncogene or tumor suppressor gene^6^. To resolve this discrepancy, we hypothesized that distinct CUX1 protein isoforms explain these disparate functions.

**Figure 1:**
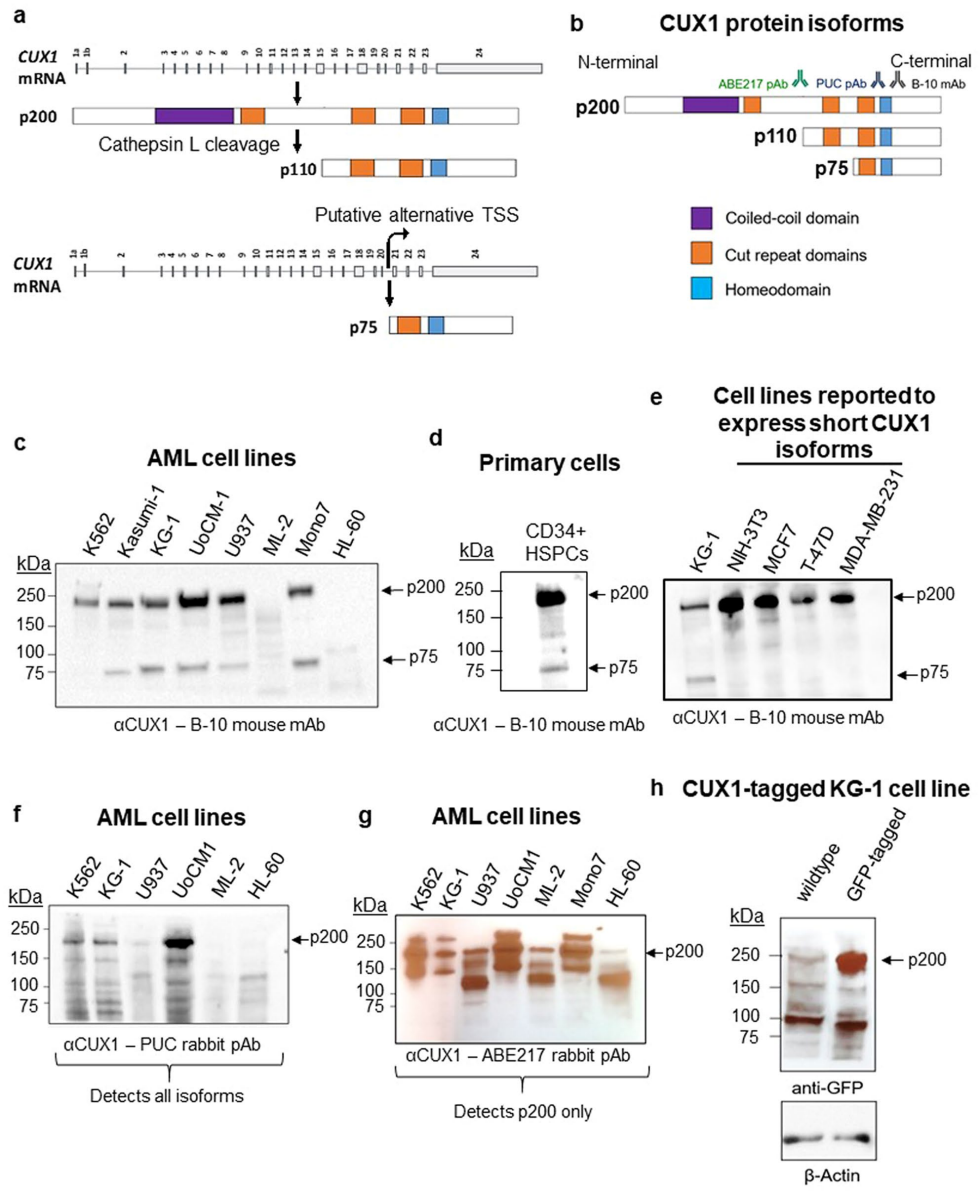
Human hematopoietic cells only express the p200 CUX1 isoform. (**a**) Schematic representation of the *CUX1* mRNA. There are two *CUX1* mRNA transcripts that vary only by the alternative first exons (1a and 1b). CUX1 encodes a full-length protein of 1505 amino acids which runs at 200 kDa (p200). A truncated p110 CUX1 protein is reported to be generated by proteolytic cleavage by cathepsin L. The p75 CUX1 isoform is reported to arise from an alternative transcription site embedded within intron 20. (**b**) Schematic representation of the predominant CUX1 protein isoforms, with protein domains indicated, and the CUX1 antibodies used in this study. (**c**) Immunoblot of CUX1 in the indicated human AML cell lines, using the B-10 antibody (n = 3). 10 μg of protein was loaded for the K562 and Kasumi-1 cell line, and 15 μg of protein was loaded for all other cell lines. (**d**) Immunoblot of CUX1 in primary human CD34 + HSPCs using the B-10-HRP antibody (n = 3). (**e**) Immunoblot of CUX1 in the NIH-3T3 fibroblast line and several human breast cancer cell lines previously reported to express p75 CUX1 using the B-10-HRP antibody (n = 3). (**f**) Immunoblot of CUX1 in indicated human AML cell lines, using the PUC antibody (n = 3). (**g**) Immunoblot of CUX1 in indicated human AML cell lines, using the ABE217 antibody (n = 3). (**h**) Immunoblot of GFP in a KG-1 cell line where endogenous CUX1 is C-terminally tagged with GFP. Protein from unedited KG-1 cells is also included (n = 3). Blot is cropped from the same gel to remove an intervening irrelevant lane.

The two RefSeq-annotated *CUX1* mRNA transcripts vary only by alternative first exons and encode a full-length protein of 1505 amino acids length, described in the literature as p200 (Figs. 1a, S1). p200 CUX1 has four DNA-binding domains, comprised of three CUT-repeat domains and one homeodomain (Fig. 1a). A truncated p110 CUX1 isoform is generated by post-translational proteolytic processing of full-length p200 CUX1 by cathepsin L (Fig. 1a)^10^. This cleavage occurs during the S phase in normal cells, and can become constitutive in transformed cells^10,11^. p110 CUX1 lacks one CUT-repeat domain and the N-terminal region but retains the three C-terminal DNA-binding domains. A third isoform, p75 CUX1, is reported to arise from an alternative transcription start site (TSS) embedded within intron 20 and retains one CUT-repeat and the homeodomain (Fig. 1a)^6,12^. p75 has been identified in human breast cancer cell lines and mouse thymocytes^12^. Despite fewer DNA binding domains, p75 and p110 bind DNA more stably than p200^11,13^. Rarer CUX1 isoforms have been described to be generated by post-translational proteolytic processing; p80, p90 and p150 CUX1^13–15^. However, these isoforms are less well characterized and it is unclear if they bind DNA and exert transcriptional activity^13–15^.

By some reports, *CUX1* is thought to be oncogenic in cancer. Over-expression of short p75 or p110 CUX1 isoforms in fibroblasts and breast cancer cells causes increased proliferation, cell cycle progression, and tumor formation in vivo^11,12,15–19^. p75 CUX1 transgenic mice engendered a higher proportion of adenosquamous mammary carcinomas and lung metastases compared to p110 or p200 transgenic mice^18^. On the other hand, large scale cancer genome resequencing efforts demonstrate that patterns of *CUX1* inactivating mutations or deletions are more characteristic of a tumor suppressor^20^. *CUX1* deletions and inactivating mutations are prevalent across cancer types^20–22^. In myeloid malignancies, *CUX1* falls within the commonly deleted region of chromosome 7q22^23^. Consistent with a tumor suppressor role, CUX1 knockdown in mouse models leads to MDS/MPN that is reminiscent of human disease^24,25^. CUX1 knockdown in human hematopoietic stem cells provides an engraftment advantage in immunodeficient mice^23^. Even in *Drosophila* models, the *CUX1* orthologue, *cut*, exerts tumor suppressive activity^21,23^.

Given the clinical significance, it is critical to parse out the putative oncogenic and tumor suppressive roles of *CUX1*. We reasoned that uncovering isoform-specific properties would reveal therapeutic strategies for inhibiting oncogenic CUX1 isoforms or promoting the expression of tumor suppressive isoforms to treat malignancies with *CUX1* alterations. To this end, we characterized the CUX1 isoforms in a panel of human cell types and leveraged publicly available functional genomic datasets across a spectrum of tissue types. To our surprise, we identified no evidence supporting the existence of the p75 mRNA isoform in any cell type examined and demonstrate that p75 is likely a western blotting artefact. Focusing on hematopoietic cells, we only identify the p200 isoform. Our data indicate that in hematopoietic cells, the tumor suppressive role of *CUX1* is attributable to the full-length protein. In addition, the lack of evidence for a p75 transcript calls into question prior interpretations of studies using exogenously overexpressed short isoforms that ascribe an oncogenic role for CUX1.

## Results

### Human hematopoietic cells only express the p200 CUX1 isoform

Given the relevance of CUX1 to myeloid malignancies, we first sought to identify the CUX1 isoforms expressed in human acute myeloid leukemia (AML) cells. Immunoblotting with an antibody that recognizes an epitope shared across all CUX1 isoforms (clone B-10, Fig. 1b) reveals six of eight AML cell lines express a dominant p200 CUX1 band (Fig. 1c). We also observed a less prominent 75 kDa band in five cell lines (Fig. 1c). p200 was also the predominant isoform in primary human CD34+ hematopoietic stem and progenitor cells (HSPCs), the normal counterpart thought to give rise to myeloid malignancies (Fig. 1d). To discern if the p75 band we observed corresponds to that described previously, we assessed cell lines reported to express p75 or p110: murine NIH-3T3 fibroblasts (p110), and MCF7, T47D, and MDA-MB-231 human breast cancer cell lines (p75)^12,26^. In contrast to prior findings, we did not observe a short isoform protein band in any of these cell lines, using both the B-10 and the PUC antibodies (Fig. 1e, S2). The absence of p110 in NIH-3T3 cells was also previously observed^12^.

We next blotted AML cell lines to determine if we could detect the p75 band with other CUX1 antibodies. We used a polyclonal antibody we previously generated, PUC, that recognizes amino acids 1223–1242 of CUX1^27^, and ABE217, a polyclonal antibody raised against an epitope spanning amino acid 861 of CUX1 (Fig. 1b). There were several background bands observed with PUC, but no dominant p75 band (Fig. 1f). The faint band at 75 kDa seen with the ABE217 antibody cannot be the p75 isoform, as the antibody recognizes an epitope of CUX1 upstream of the p75 protein sequence (Fig. 1b, g). Thus, p200 is the predominant CUX1 isoform in human hematopoietic cells, and we did not detect p75 or p110 in cells previously reported to express short isoforms.

To circumvent potential antibody artefacts, we took an alternative approach to determine if the p75 band is in fact CUX1, by tagging the endogenous *CUX1* allele with an in-frame C-terminal GFP tag by CRISPR/Cas9 homology-mediated repair. We used KG-1 cells, which express both p75 and p200 bands. KG-1 cells have a partial deletion of chromosome arm 7q that includes *CUX1*, thus they are mono-allelic for *CUX1,* enabling facile CRISPR/Cas9 editing (Fig. S3). GFP-tagged CUX1 migrated at a higher molecular weight than endogenous CUX1, as expected (Fig. S4). Probing these cells with an anti-GFP antibody only identified a single unique p200 CUX1 band in the tagged cell line (Fig. 1h). Together, these data indicate that human AML and primary HSPCs express p200 and not shorter CUX1 isoforms.

### CRISPR/Cas9 genomic editing precludes the existence of a CUX1 p75 isoform

**Figure 2:**
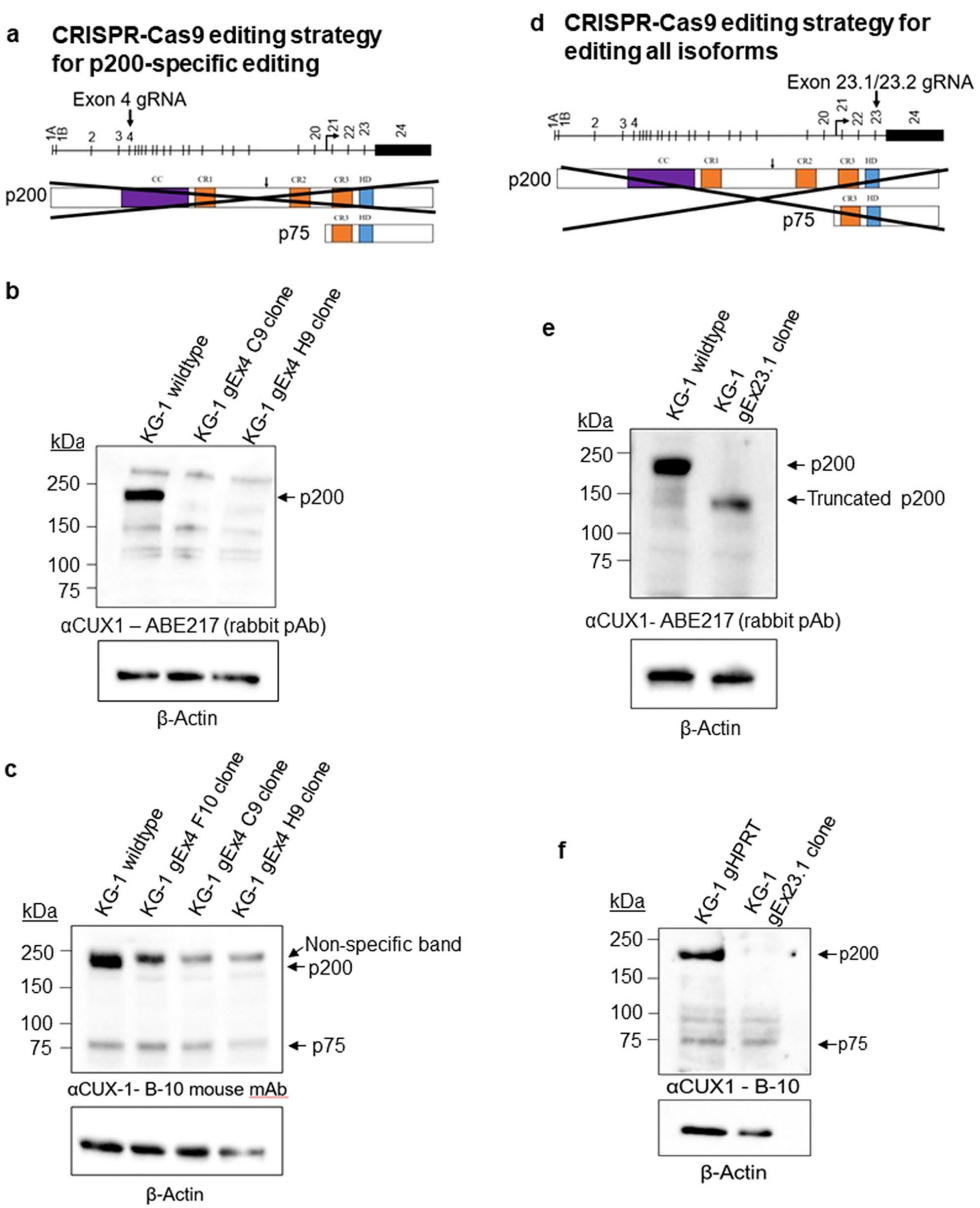
CRISPR/Cas9 genomic editing precludes the existence of a p75 CUX1 isoform. (**a**) CRISPR/Cas9 editing approach to selectively delete p200 in the KG-1 cell line, which only has one copy of *CUX1*. (**b**) Immunoblot for CUX1 in KG-1 single cell clones edited with a gRNA targeting exon 4 of *CUX1* using the ABE217 antibody. (**c**) Immunoblot for CUX1 in KG-1 single cell clones edited with a gRNA targeting exon 4 of *CUX1* using the B-10-HRP antibody. (**d**) CRISPR/Cas9 editing approach targeting exon 23 of CUX1 to delete all CUX1 isoforms in the KG-1 cell line. (**e**) Immunoblot for CUX1 in a KG-1 single cell clone edited with a gRNA targeting exon 23 of *CUX1* using the ABE217 antibody (n = 3). (**f**) Immunoblot for CUX1 in a KG-1 single cell clone edited with a gRNA targeting exon 23 of *CUX1* or *HPRT* using the B-10 antibody (n = 3).

**Figure 3:**
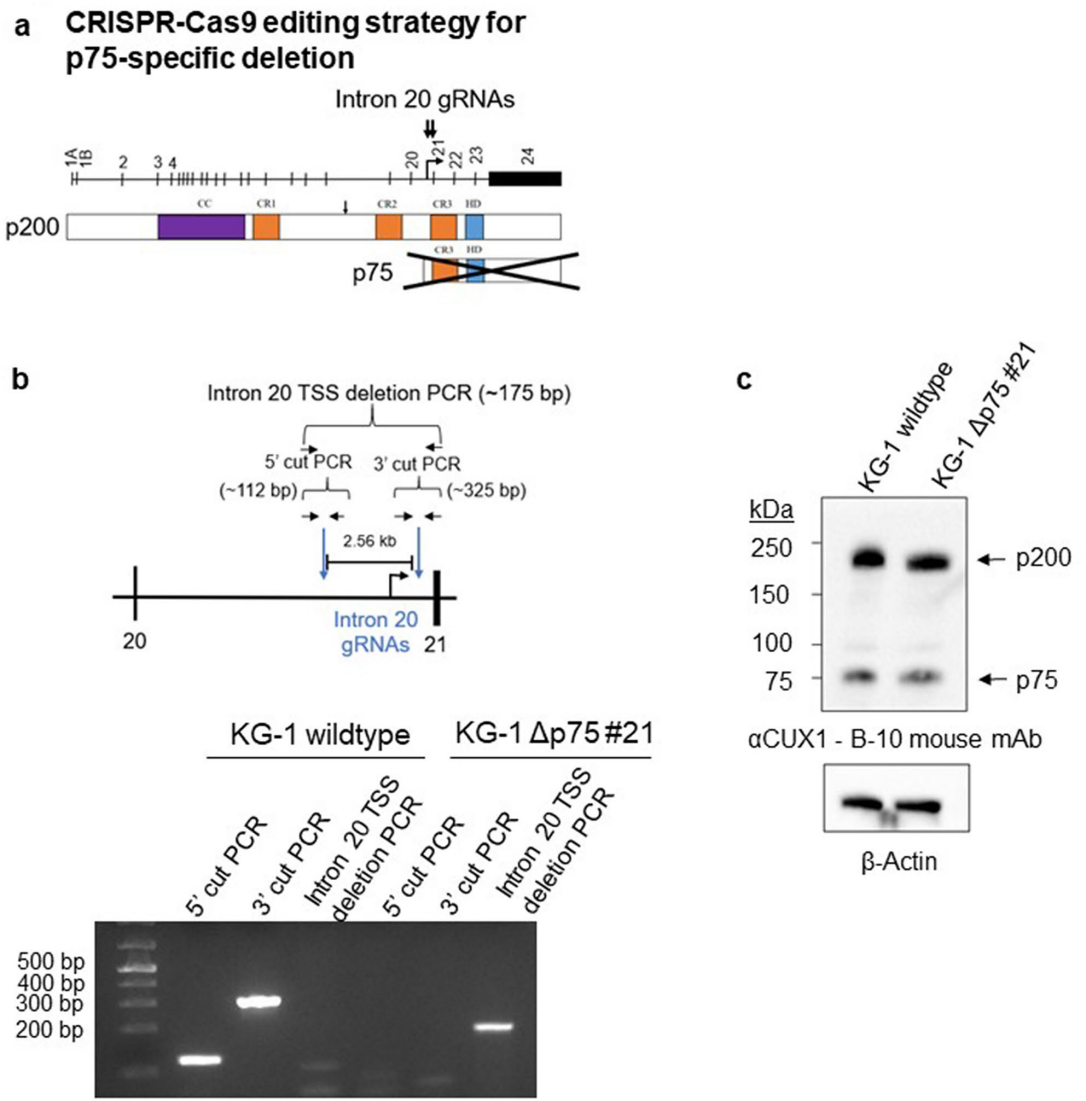
CRISPR/Cas9 genomic editing of the predicted intronic p75 transcriptional start site does not eliminate the p75 isoform band. (**a**) CRISPR/Cas9 editing approach using two gRNAs flanking the predicted intronic p75 TSS. (**b**) PCR screening strategy for identifying single cell clones with deletion of the p75 putative TSS. PCR products from primer pairs spanning the first cut site or the second cut site in intron 20 indicate the lack of a successful deletion. A PCR product from primers spanning the deletion site indicates a successful deletion. (**c**) Immunoblot for CUX1 using the B-10 antibody in a KG-1 single cell clone with successful deletion of the p75 TSS (n = 3).

To further interrogate if the p75 band is encoded by the *CUX1* locus or is a non-specific western blotting artefact, we devised several CRISPR/Cas9 strategies to selectively target KG-1 genomic DNA encoding p75, p200, or both (Figs. 2a, d, 3a). As illustrated in Fig. 2a, we designed a gRNA targeting exon 4 of *CUX1* which is only expressed in the genomic region encoding the p200 isoform. This would be expected to selectively introduce a frameshift mutation in the p200 protein only and thereby induce nonsense-mediated decay of only p200, leaving p75 intact. We identified two single-cell CRISPR-edited clones that had a complete loss of p200 CUX1 (C9 and H9), best appreciated with the ABE217 antibody (Fig. 2b). The B-10 antibody also shows a loss of p200, with a residual non-specific band migrating at a slightly higher molecular weight (Fig. 2c). The expression level of the p75 band was unchanged, as expected based on our targeting strategy (Fig. 2c).

As a control, we designed a gRNA targeting exon 23 of *CUX1* (gEx23.1) to introduce frameshift mutations in and edit all *CUX1* transcripts (Fig. 2d). As exon 23 is shared by all isoforms, including p75, it would be expected to disrupt all bona fide CUX1 proteins. Transfection with gEx23.1 followed by single cell cloning identified a clone with a single base pair insertion generating a frameshift mutation. We blotted the gEx23.1-edited KG-1 clone with ABE217 and observed that the band for p200 CUX1 shifted downward, consistent with a predicted C-terminal truncation of ~ 28 kDa (Fig. 2e). The B-10 antibody binds an epitope after the exon 23 gRNA cut site, thus all gEx23-edited isoforms will be undetectable with B-10 (Fig. 1b). Indeed, probing with B-10 demonstrates abolished expression of the p200 CUX1 band, yet persistent p75 (Fig. 2f). The fact that the 75 kDa band remained indicates that it is not encoded by the *CUX1* locus.

We similarly targeted *CUX1* using two different exon 23 gRNAs in primary human CD34+ HSPCs, and saw no change in expression of any bands other than p200 CUX1 (Fig. S5). The residual p200 protein in the gEx23 edited lanes is consistent with ~ 75–80% editing in these bulk populations. Overall, these data indicate that hematopoietic cells express p200 CUX1 and not p75.

We considered the possibility that p75 does not contain exon 23, perhaps due to alternative splicing. As a different approach, we designed a pair of gRNAs flanking the predicted p75 intronic TSS reported to be ~ 2.5 kb upstream of exon 21 (Fig. 3a)^12^. We reasoned that eliminating the putative intronic TSS would eliminate transcription of the p75 isoform while leaving p200 unperturbed. We deleted approximately 2.56 kb of intronic DNA, leaving 79 base pairs intact proximal to exon 21. Using a PCR strategy to screen the expected deletion, we generated a successfully deleted single-cell clone (KG-1 Δp75 #21, Fig. 3b). Immunoblotting, however, indicated no change in p75 (Fig. 3c). This suggests that this 2.56 kb segment of DNA does not harbor the putative p75 TSS, and is incongruent with the prior report^12^. In summary, these experiments show that the presumptive p75 isoform contains neither exon 23 nor a TSS within 2.56 kb upstream of exon 21 as originally described, further implicating the p75 band as a western blotting artefact.

### Proteomics approaches do not support the existence of p75 CUX1

**Figure 4:**
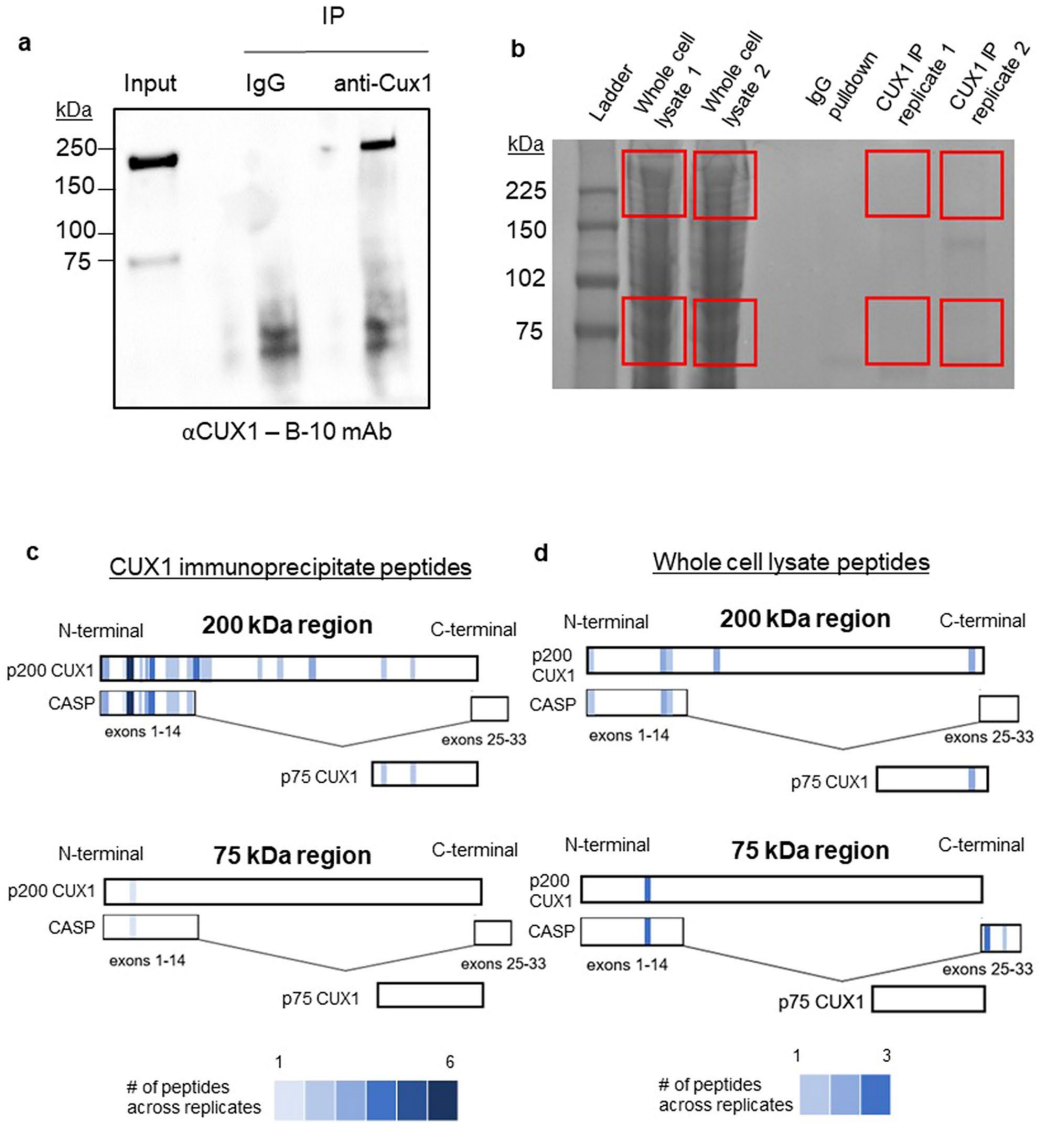
Proteomic approaches do not support the existence of p75 CUX1. (**a**) B-10 immunoblot after B-10 immunoprecipitation of CUX1 in the KG-1 cell line. KG-1 cells were also immunoprecipitated with a mouse IgG antibody as a negative control. Input control is also included (n = 3). Blot is cropped on the edge to remove a redundant lane. (**b**) p200 and p75 regions (red rectangles) were excised from Imperial stained SDS/PAGE of two whole cell extract replicates in the KG-1 cell line, and two B-10 immunoprecipitated CUX1 replicates from the KG-1 cell line. (**c**) Schematic showing the number and distribution of peptides mapping to CUX1 and CASP protein sequences from the immunoprecipitated CUX1 samples (n = 2). Heat map at the bottom depicts the number of each peptide detected across replicates. Peptides with ambiguous assignments are shown in both potential candidate isoforms. (**d**) Schematic showing the number and distribution of peptides mapping to CUX1 and CASP protein sequences from the whole cell lysate samples. Heat map at the bottom depicts the number of each peptide detected across replicates. Peptides with ambiguous assignments are shown in both potential candidate isoforms.

We considered that the p75 artefact results from the denaturing conditions of western blotting. To test this, we performed a CUX1 immunoprecipitation which is performed with proteins in their native state. After immunoprecipitation of CUX1 in KG-1 cells with the B-10 antibody, we probed the resulting blots again with B-10. While the p75 band is present in the input control, we do not observe it in the immunoprecipitate (Fig. 4a). This result is consistent with p75 being an artefact of western blotting.

We turned to unbiased proteomics to search for any CUX1 peptides within the 75 kDa region. We subjected KG-1 whole cell lysates and CUX1 immunoprecipitates to SDS-PAGE. We excised regions corresponding to 200 kDa and 75 kDa and performed LC–MS/MS (Fig. 4b). In the 200 kDa region after CUX1 immunoprecipitation, we observed 51 peptides (21 unique, shown as blue rectangles, Fig. 4c; Table S1) summed across replicates that mapped to CUX1/CASP protein sequences. Because p200, p75 and CASP partially share exons, some peptides are ambiguous and thus map to both p200 and CASP or both p200 and p75. However, as CASP is a 77 kDa protein and p75 is 75 kDa, we can infer that all ambiguous peptides in the 200 kDa region are in fact p200. In the 75 kDa region of the immunoprecipitated samples, we observed one peptide that ambiguously mapped to the N-terminal region of CUX1/CASP, but observed no peptides mapping to the p75 region (Fig. 4c; Table S2). We think this might be a degradation product from the p200 band for this sample, as it was a low-scoring peptide of low intensity, compared to the corresponding peptide in the p200 sample. These data confirm that p75 CUX1 does not immunoprecipitate with anti-CUX1, consistent with Fig. 4a.

We assessed the proteomic analysis of whole cell lysates, which are agnostic to antibody selection (Fig. 4b, first two lanes). The p200 band contained 8 peptides (5 unique) which, based on a priori knowledge of relative protein migrations, we ascribed to p200 (Fig. 4d; Table S3). Within the p75 band isolated from lysates, we observed 7 peptides (3 unique) that all unambiguously map to CASP. We did not detect any peptides from the p75 region in the lysates that mapped to p75 CUX1 (Fig. 4d; Table S4). Taken together, we conclude that there is no proteomic evidence of the p75 isoform in a representative AML cell line that possesses the p75 protein band.

### No p75 *CUX1* is detected at the RNA level in human AML and breast cancer cell lines

**Figure 5:**
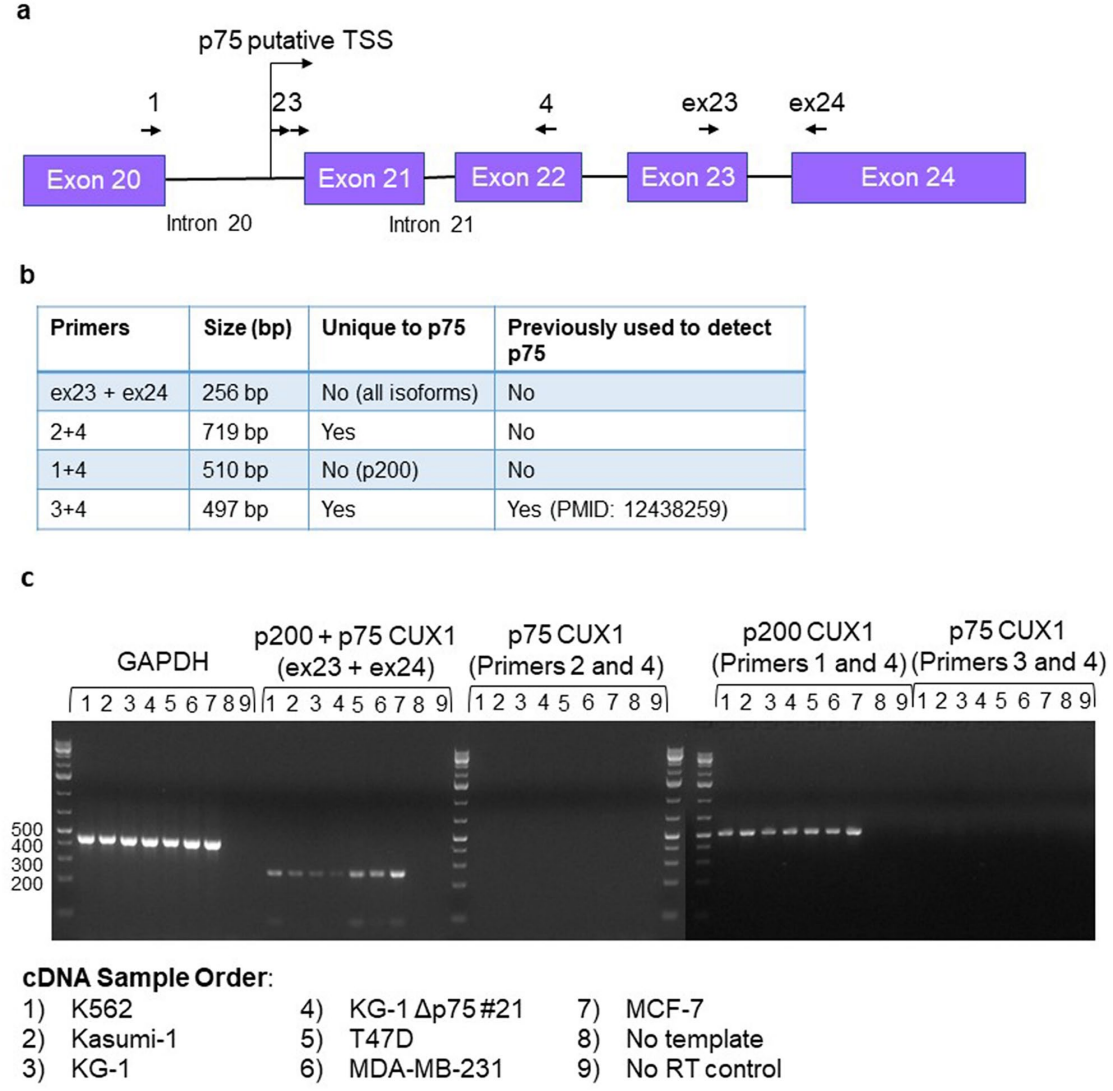
No p75 CUX1 is detected at the RNA level in human AML and breast cancer cell lines. (**a**) Schematic of primers used to detect p75. (**b**) Table of anticipated PCR products and whether they are unique to the p75 isoform. (**c**) Reverse-transcriptase PCR products after 30 cycles of PCR spanning the intron 20 region of CUX1 using cDNA reverse-transcribed from mRNA in 6 different cell lines (K562, Kasumi-1, KG-1, T47D, MDA-MB-231 and MCF-7) (n = 3). cDNA from the KG-1 Δp75 #21 cell line was used as a negative control for p75 mRNA expression. *GAPDH*, p200-specific primers (1 and 4) and *CUX1* primers spanning all isoforms (ex23 and 24) were used as positive controls. Gel is cropped for clarity.

It remained possible that the p75 protein is below the level of detection of mass spectrometry. As a more sensitive test, we looked for the presence of p75 *CUX1* at the mRNA level by RT-PCR, using two primer sets (pairs 2 and 4 and pair 3 and 4) spanning intron 20 and exon 22 (Fig. 5a, b). Primer pair 3 and 4 was previously reported to detect the p75 transcript^12^. We assessed cDNA from three AML cell lines (K562, Kasumi-1, and KG-1) and three human breast cancer cell lines described to express p75 (T47D, MDA-MB-231, and MCF-7). Primers for *GAPDH* and all *CUX1* isoforms (primer pair 1 and 4 and pair ex23 and ex24) served as positive controls. We did not detect any bands with the previously reported p75 primers (3 and 4) or with our primers (2 and 4, Fig. 5c). We confirmed that primer 4 was functional, as it successfully amplified p200 (primer pair 1 and 4), indicating that the lack of a p75 transcript was not due to primer design issues. Overall, we do not detect a p75 transcript in either human AML cell lines or the breast cancer cell lines previously reported to express p75^12^.

### Functional genomics consortia datasets lack epigenetic or transcriptional evidence for a CUX1 p75 intronic transcriptional start site

**Figure 6:**
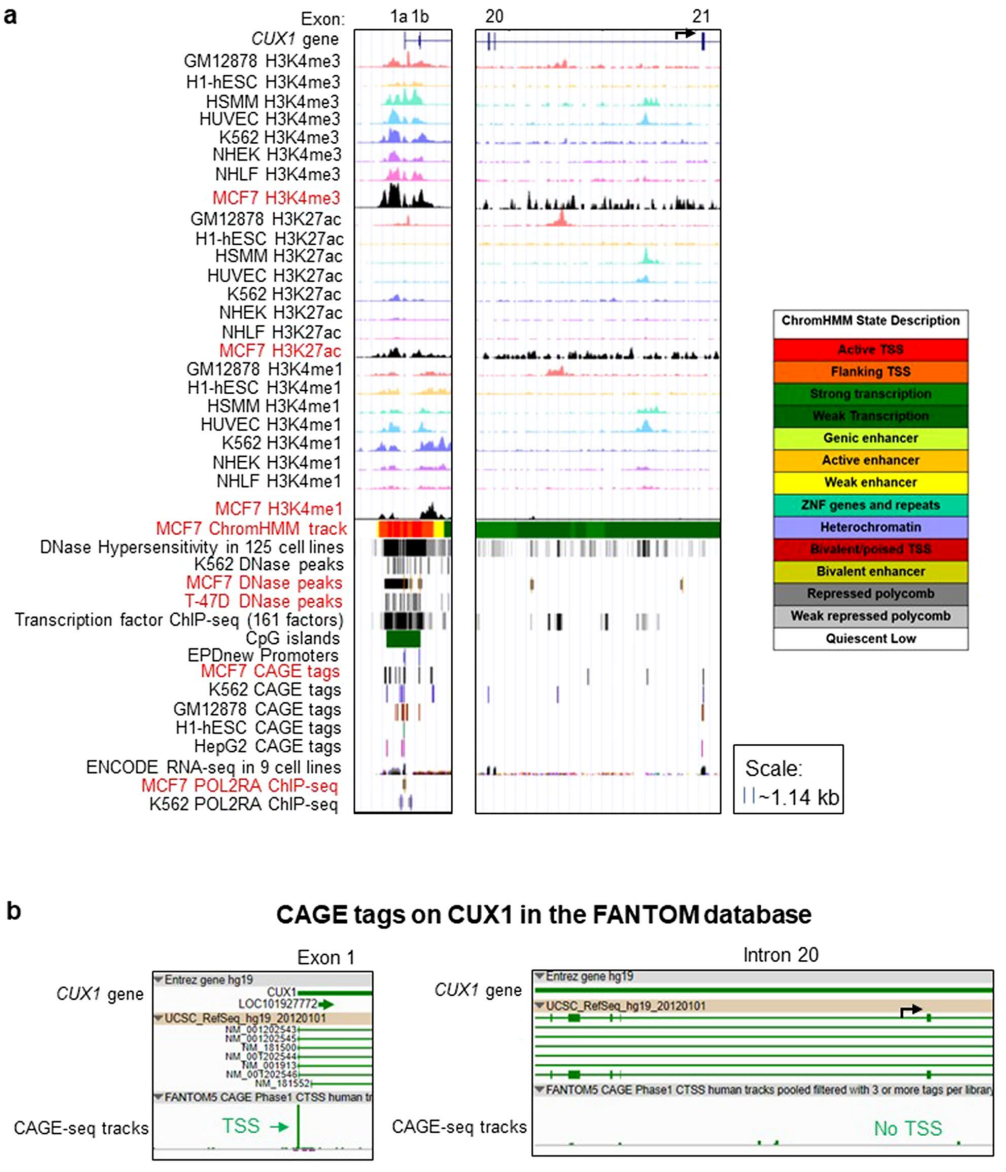

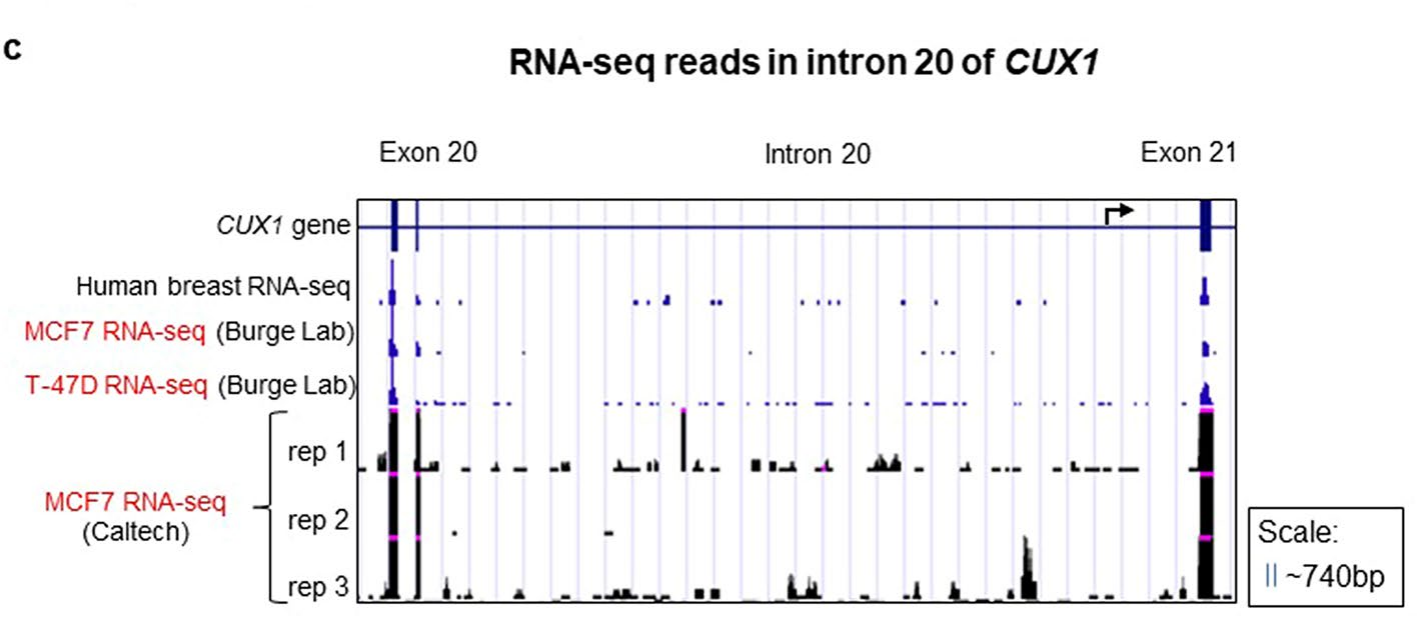
Functional genomics consortia datasets lack epigenetic or transcriptional evidence for a CUX1 p75 intronic transcriptional start site. (**a**) CUX1 Refseq gene model is aligned with tracks of indicated transcriptional and epigenetic marks (including H3K4me1, H3K4me3 and H3K27ac ChIP-seq, DNase hypersensitivity tracks, CAGE-seq data in 5 ENCODE cell lines, POL2RA ChIP-seq tracks, and transcription factor ChIP-seq tracks). Tracks highlighted in red are derived from a cell type previously reported to express p75 CUX1. The ENCODE RNA-seq data in 9 cell lines was overlaid using the Transparent setting in the same track. (**b**) CAGE-seq data from the FANTOM browser at exon 1 and intron 20 of CUX1 in 975 human primary cells and cancer cell lines. (**c**) RNA-seq peaks for *CUX1* expression in human breast tissue, MCF7 and T-47D cell lines from Burge lab sequencing data^37^ visualized on the UCSC Genome Browser. No intronic reads contiguous with exon 21 are observed.

Heretofore, our analysis has encompassed thirteen cell types. We sought to extend our analysis to comprehensively assess additional normal and malignant tissue types. As such, we leveraged consortia-generated functional genomics datasets for evidence of p75 CUX1 across a variety of cell types. We first assessed epigenetic marks that canonically decorate promoters. Promoters and enhancers both have H3K27ac, H3K4me3 and H3K4me1 deposition, although enhancers have higher levels of H3K4me1^28^. All three of these marks are present at the p200 TSS across seven Tier 1 ENCODE cell lines (Fig. 6a). However, we did not observe any H3K4me3 peaks in the intron 20 region of CUX1, including in MCF7, previously reported to express p75 (Fig. 6a). We observed some H3K27ac and H3K4me1 peaks in intron 20, but these peaks were not at the predicted TSS and not in MCF7 cells (Fig. 6a). Additionally, we also looked at the ChromHMM track in MCF7 cells. ChromHMM is a computational approach that integrates experimental ChIP-seq datasets for different histone marks into a hidden Markov model to assign chromatin states genome-wide^29^. The MCF7 ChromHMM track shows an active TSS (red region) at exon 1a and 1b but only weak transcription (dark green) and no promoter regions in the intron 20 region (MCF7 ChromHMM track, Fig. 6a). Thus, epigenetic features of promoters are not present at the putative p75 TSS, even in a cell type documented to express p75.

Promoters are also characterized by accessible chromatin, transcription factor binding, RNA-polymerase II (POL2RA) occupancy, and CpG islands^30–33^. In line with this, the p200 TSS has pronounced DNase hypersensitivity, transcription factor occupancy, POLR2A peaks, and a CpG island (Fig. 6a). However, there is a paucity of these signals in intron 20 and they are not enriched in the previously mapped p75 TSS. There is one DNase accessibility site in MCF7 near the predicted p75 TSS, but it does not have any of the other features of a promoter. These data are all inconsistent with an intron 20 alternative TSS.

Efforts have been made to comprehensively annotate promoters by synthesizing functional genomic datasets and curated databases. One such catalog is the Eukaryotic Promoter Database (EPD), which correctly annotates both exon 1a and 1b but not any promoters within intron 20 (Fig. 6a “EPDnew Promoters” track)^34^.

We confirmed this observation with next-generation sequencing-based cap-analysis gene expression (CAGE-seq), which captures 5′ capped mRNA transcripts to map TSSs^35^. CAGE-seq identifies 5′ capped mRNA in 5 different ENCODE cell lines (GM12878, H1-hESC, K562, HepG2 and MCF7) at exons 1a and 1b of *CUX1* yet none within intron 20, including in MCF7 (Fig. 6a). Similar results were observed from CAGE-seq data from an atlas of 975 human primary cells and cancer cell lines (Fig. 6b)^36^. In aggregate, these data do not support an alternative intronic TSS.

The presumptive p75 transcript expresses intronic sequence proximal to exon 21^12^. We mined RNA-seq datasets to identify reads spanning this region in MCF7 and T-47D human breast cancer cell lines reported to express p75^6,12,37^. All exon 21 sequencing reads abruptly end at the intron 20/exon 21 border, inconsistent with the p75 transcript (Fig. 6c). In the MCF7 cell line, we observed some sequencing reads within the intron 20 region, but these were not conserved across four different replicates and were not contiguous with exon 21 reads^37^ (Fig. 6c). These data do not support the existence of a p75 mRNA containing intronic sequence.

Finally, we note that no gene assembly or gene prediction database for coding or non-coding RNA annotate a p75 isoform. NCBI Refseq, CCDS, GENCODE, Ensembl gene predictions, AUGUSTUS, or ORFeome all lack a p75 transcript (Fig. S6)^38–42^. Collectively, these extensive datasets spanning thousands of samples provide no evidence of a p75 CUX1 transcript.

## Discussion

Next-generation sequencing and CRISPR/Cas9 genome editing has revolutionized biomedical research in many ways. Perhaps less appreciated, however, is the role of these technologies in establishing the legitimacy of research findings. CRISPR/Cas9 editing, for instance, has invalidated cancer dependencies, drug targets, and viral receptors, as some illustrative examples^43–45^. In this report, we employ the power of functional genomics and CRISPR editing to demonstrate that the *CUX1* gene does not encode a p75 isoform as described^12^. This conclusion is buttressed via multiple orthogonal approaches including biochemical studies with several antibodies, proteomics and extensive mining of functional genomic datasets across a plethora of cell types. Taken together, our data is inconsistent with a p75 CUX1 isoform arising from an intronic TSS and suggests that prior reports were based on a western blotting artefact^12^.

Other studies support our conclusion. p75 was first identified in HeLa cells, HEK293 cells, breast cancer cell lines and mouse thymus^12^. The original manuscript identifying human CUX1 generated antiserum against the entire CUX1 protein, yet western blotting of HeLa cells identified only p200^46^. Probing HEK293 cells with an antibody against the C-terminus region of CUX1 shows no p75 in another study^19^. In a report that p75 CUX1 causes polycystic kidney disease, the endogenous p75 expression was never documented at the protein level; all subsequent experiments were performed by over-expressing p75 cDNA^47^. Probing a western blot of entire *Drosophila* embryos for the highly conserved CUX1 ortholog, Cut, only reveals the full-length protein^48^. While p200 CUX1 protein increases after TGF-β treatment in normal lung fibroblasts, p75 does not^49^. Finally, in a study that reported that androgen-resistant prostate cancer cell lines upregulate p200, p75 was unchanged^50^. Collectively, these studies either fail to document an endogenous p75 protein, or uncouple the biology of p200 from p75.

It is unclear what the p75 cDNA product previously reported represents^12^. Perhaps it is a result of recursive splicing, where long introns are spliced in a sequential manner in tissue-specific contexts leading to a lag in splicing of intron 20 material^51^. Alternatively, studies of nascent transcription indicate that splicing does not always occur in the order of transcription, and introns that are spliced later temporally tend to be longer and have higher RNA-binding protein occupancy^52^. In keeping with this notion, intron 20 is the sixth longest intron in *CUX1*, and has an elevated RNA-binding protein occupancy compared to most other introns in the gene and thus may be spliced later than other introns in *CUX1*^52^. It is conceivable that the p75 cDNA product previously observed represents an intermediate, incompletely spliced p200 cDNA.

In our analysis of hematopoietic cells, we only document the expression of the p200 CUX1 isoform. We cannot comment on the validity of p110 or other short isoforms (p80, p90 and p150). As these isoforms are generated post-translationally, functional genomics datasets are ineffective in determining their legitimacy. Future studies of these putative isoforms in other tissue types should employ stringent techniques such as CRISPR/Cas9 mutagenesis to ensure against being misled by western blotting artefacts.

Our finding calls into question studies that ascribed oncogenic functions to p75 CUX1. Many of these publications did not study endogenous p75, but instead employed overexpression models, which can confound results^12,18,19,53,54^. We speculate that overexpression of CUX1 has dominant negative effects. For instance, in mice, both overexpression of p75 and knockout or knockdown of CUX1 leads to myeloproliferative disease^19,24,55^. One interpretation of these seemingly incongruent findings is that artificial overexpression of p75 either interferes with the stoichiometry of endogenous CUX1 protein complexes or blocks full-length CUX1 from binding to its target genes. Indeed, p75 CUX1 has increased DNA-binding affinity compared to endogenous p200 CUX1^56^. The net effect of CUX1 overexpression may be the disruption of endogenous CUX1 tumor suppressor activity. In support of this model, the p150 CUX1 isoform was found to exert a dominant negative phenotype upon p200 CUX1^14^. In this light, the use of overexpression systems to characterize p110 may also misattribute this isoform with oncogenic properties^16,18,53,57,58^.

There are relatively fewer reports that p200 CUX1 is oncogenic. p200 CUX1 has been shown to promote cell line migration, invasion, and evasion of apoptosis^17,59^. p200 transgenic mice develop organ hyperplasia^60^. 7q copy number gains and CUX1 overexpression has been documented in primary cancers^6,61,62^. However, these later findings should be interpreted with caution. Chromosome 7 also encodes oncogenes, including *EGFR* (on 7p), and *BRAF*, *CDK6,* and *EZH2* (on 7q). Thus, in cancers with chromosome 7 copy number gains, the driver may be a true oncogene, while *CUX1* is a passenger. Indeed, rigorous pan-cancer gene-level analysis of copy number alterations and mutation patterns in primary patient samples reveal *CUX1* genetic changes are significantly characteristic of a tumor suppressor gene^20–22^. There is now a growing body of work that CUX1 is tumor suppressive^21,23–25,27,63^.

Given the importance of CUX1 in development and disease across a wide variety of tissue types, it is critical to carefully dissect and understand the genomic structure of the *CUX1* locus and encoded protein. The complexity of the gene has led to confusion in the field resulting in serious inaccuracies, most recently by Xu, et. al.^64^. We expect that our current study will help rectify these obstacles going forward.

## Methods

### Human cell culture

KG-1, Mono7, ML-2, HL-60, Kasumi-1, and UoCM1 were grown at 37 °C in Gibco RPMI 1640 media supplemented with 20% fetal bovine serum (FBS) and 1X antibiotic–antimycotic (Gibco). K562, U937 and T-47D cell lines were grown in Gibco RPMI 1640 media supplemented with 10% FBS and 1X antibiotic–antimycotic. NIH-3T3 fibroblasts, MCF7 and MDA-MB-231 breast cancer cell lines were grown in Gibco DMEM media supplemented with 10% FBS and 1X antibiotic–antimycotic. All cell lines were verified by STR analysis at the NUSeq Core Facility at Northwestern University.

Human mobilized peripheral blood CD34+ HSPCs from multiple healthy donors were purchased from the Fred Hutchinson Co-operative Center for Excellence in Hematology (Seattle, WA, USA). CD34+ HSPCs were expanded in StemSpan SFEMII base media supplemented with CC110 culture supplement for 1–3 days prior to electroporation.

### Generation of CUX1-GFP tagged KG-1 cell line

pCUX1.1.0-gDNA (Addgene plasmid #112434; RRID: Addgene_112434) and pCUX1-donor plasmids (Addgene plasmid #112338; RRID: Addgene_112338) were a gift from Kevin White. The *CUX1* homology arms were chemically synthesized by Gibson assembly and comprised 0.6 kb upstream and 1 kb downstream *CUX1* sequence flanking the stop codon. The GFP tag is a LAP tag that contains a TEV protease cleavage site, an S peptide, the EGFP coding sequence, followed by an IRES and a kanamycin resistance element^65^. The LAP-GFP tag was PCR amplified from a separate plasmid, and assembled with the CUX1 donor sequence using Gibson assembly. KG-1 cells were transfected with 0.5 μg of each plasmid using the Neon® Transfection System (Invitrogen by Life Technologies, Waltham, MA, USA). The electroporation settings used for transfection was 1650 V, 20 ms pulse width, 1 pulse, and electroporation was performed according to manufacturer instructions. Cells were cultured in 0.5 mg/ml G418 after 7 days for 3 weeks to select for a transfected population. Primers used to confirm correct integration of the GFP tag are listed below:

- *EGFP* 5′ primer: 5′-CATGAAGCAGCACGACTTCT-3′.
- *EGFP* 3′ primer: 5′-CTGCTTGTCGGCCATGATATAG-3′.
- 5′ primer spanning homology arm and *EGFP*: 5′-GGAACCTATCGAATGGGAGTTC-3′.
- 3′ primer spanning homology arm and *EGFP*: 5′-AAGTCGTGCTGCTTCATGT-3′.

### Immunoblots

Cells were lysed in RIPA buffer (50 mM Tris–HCl, 150 mM NaCl, 0.5% sodium deoxycholate, 0.1% SDS, 5 mM EDTA and 1% NP-40 substitute). 1% Halt™ Protease Inhibitor Cocktail (Thermo Fisher) was added to the lysis buffer before use. The lysates were passed through a 25-gauge needle, incubated on ice for 20 min, with frequent vortexing, and clarified by centrifugation (5000 g, 10 min at 4 °C). Total protein of the resulting supernatant was quantified using the Bradford assay at 595 nm wavelength, with BSA used to generate the standard curve. 10–15 µg of protein was subjected to SDS-PAGE and probed with anti-CUX1 antibody conjugated to HRP (B-10-HRP, mouse mAb derived against aa 1308–1332, Santa Cruz, 1:1000 in 5% milk/TBST) and visualized using ECL substrate. β-Actin was detected with anti-β-actin-HRP (C4, Santa Cruz, 1:3000 in 5% milk/TBST). Other antibodies used to probe for CUX1 expression include ABE217 (rabbit polyclonal antibody derived against aa 861, 1:1000 in 5% milk/TBST), and PUC (rabbit polyclonal antibody generated in-house that recognizes aa 1223–1242, 1:1000 in 5% milk/TBST). GFP (D5.1) rabbit mAb #2956 (Cell Signaling Technology, Product #2956S, 1:1000 in 5% FBS/TBST) was used to probe for GFP-tagged CUX1. Nitrocellulose membrane (Thermo Scientific, Catalog number: 88018) was used for protein transfer and ECL substrate (Thermo Scientific, Catalog number: 34579) was used for visualizing the proteins of interest.

### gRNA design

All gRNAs were designed using the Broad Institute’s sgRNA designer tool, and generated gRNA sequences were verified using Synthego’s Verify Guide Design tool. gRNA sequences used in this study were purchased from Synthego, and the sequences are listed below:

- *CUX1* exon 4 gRNA: 5′-UGCACUGAGUAAAAGAAGCA-3′.
- *CUX1* intron 20 5′ gRNA 1: 5′-GUAUUUCACGAUUCAGCCAA-3′.
- *CUX1* intron 20 3′ gRNA 1: 5′-CUUUGGGUCAUACAUUGGCA-3′.
- *CUX1* intron 20 5′ gRNA 2: 5′-AUGGCACAAAUCCACGCCAC-3′.
- *CUX1* intron 20 3′ gRNA 2: 5′-AUACUAAUUAAACGCUCUGU-3′.
- *CUX1* exon 23.1 (NLS) gRNA: 5′-GCUGUGCCGCCGCUUCAUGU-3′.
- *CUX1* exon 23.2 (HD) gRNA: 5′-CCAGCUGAAGAAACCCCGG-3′.
- *HPRT* gRNA: 5′-GCAUUUCUCAGUCCUAACA-3′.

### gRNA transfections

gRNA:Cas9 ribonucleoprotein (RNP) complexes were formed by mixing 1.8 μL of each gRNA at 100 μM and 1.5 μL Cas9 nuclease at 20 μM in 15 μL of electroporation buffer R. RNP complexes were incubated at room temperature for 10 min. 2 × 10^5^ KG-1 cells were pelleted and washed twice with PBS. The cells were then suspended in the RNP complex, and electroporation was performed using the Neon^®^ Transfection System (Invitrogen by Life Technologies, Waltham, MA, USA). The optimized electroporation settings used for transfecting the KG-1 cell line was 1700 V, 20 ms pulse width, 1 pulse, and electroporation was performed according to manufacturer instructions. For CD34+ HSPCs, 0.71 uL Cas9 nuclease at 20uM was mixed with 2.39 uL gRNA at 30 uM and 0.9 uL of electroporation buffer T. RNP complexes were formed by incubating for at least 15 min at room temperature. 200,000 CD34+ cells in 8 uL of Buffer T were then added to the RNPs, and 10ul of the mixture electroporated and immediately cultured in SFEMII + CC110. AAVS1 gRNA was used as a negative control. Electroporation settings used for CD34 + HSPCs was 1600 V, 10 ms pulse length, 3 pulses.

To examine the clonality of transfected cell lines, a serial dilution approach was adapted from Corning Life Sciences. Edited single cell clones were also established by sorting single cells into a 96-well plate on the AriaIIIu cell sorter.

### PCR confirmation of gRNA editing

The following primers were used to amplify the gRNA cut site from genomic DNA to verify editing efficiency:

- *CUX1* intron 20 5′ primer for first cut site: 5′-AGCGCCCCTGTTTAGTTCTC-3′.
- *CUX1* intron 20 3′ primer for first cut site: 5′-GAGCCACCACGAAACTCAGA-3′.
- *CUX1* intron 20 5′ primer for second cut site: 5′-CCCTCATGTTAAGCCTTCCGA-3′.
- *CUX1* intron 20 3′ primer for second cut site: 5′-CACTGGAACTCATCGGGGAC-3′.
- *CUX1* exon 4 gRNA 5′ primer: 5′-CCCTCCTAGACCCTGAGCTT-3′.
- *CUX1* exon 4 gRNA 3′ primer: 5′-TTCATGTGTCCTGCACTCCC-3′.
- *CUX1* exon 23 gRNA 5′ primer: 5′-GGTGAGGACCTGACATTCCG-3′.
- *CUX1* exon 23 gRNA 3′ primer: 5′-GGACCAAGGAACGGACCAAT-3′.

### Reverse-transcriptase PCR

RNA was extracted from 500,000 cells of the K562, Kasumi-1, KG-1, T47D, MDA-MB-231 and MCF-7 cell lines using Trizol, precipitated using chloroform and 70% ethanol, and purified using the RNeasy Mini kit (QIAGEN cat no 74104). 500 ng of RNA from each cell line was used to synthesize cDNA using the Thermo Scientific Maxima™ H Minus cDNA Synthesis Master Mix Kit (Thermo Scientific cat no M1661). 1 µL of this synthesized cDNA was then used in the qPCR reaction. Primers were used specific to the p200 *CUX1* isoform, the p75 *CUX1* isoform, and GAPDH primers as a housekeeping control. The primers for the p75 transcript were obtained from the paper that originally described the existence of this isoform^12^. In addition, we also designed our own primers spanning various regions of intron 20 of CUX1. Controls tested include a no template water control, and a no RT enzyme control. 30 cycles of PCR were performed. Primer sequences are listed below:

- *GAPDH* forward primer: 5′-ACCACAGTCCATGCCATCAC-3′.
- *GAPDH* reverse primer: 5′-TCCACCACCCTGTTGCTGTA-3′.
- p200 + p75 *CUX1* forward primer: 5′-CCGGAGGAGAAGGAGGCGCT-3′.
- p200 + p75 *CUX1* reverse primer: 5′-AGCTGTCGCCCTCCGAGCTG-3′.
- *CUX1* forward primer (primer 1): 5′-CCACTCCGTGACATCGCTC-3′.
- p75 *CUX1* forward primer (primer 2): 5′-CCCTCATGTTAAGCCTTCCGA-3′.
- p75 *CUX1* forward primer (primer 3): 5′-GCTATTTTCAGGCACGGTTTCTC-3′.
- p75 *CUX1* reverse primer (primer 4): 5′-TCCACATTGTTGGGGTCGTTC-3′.

### Immunoprecipitation

CUX1 was immunoprecipitated from the KG-1 cell line, and then blotted for CUX1 again to query whether short CUX1 isoforms were able to be pulled down by the anti-CUX1 antibody. 100 × 10^6^ cells were spun down for a CUX1 pulldown and a control IgG pulldown each. Cells were lysed in hypotonic buffer (5 mM EDTA, 5 mM EGTA, 5 mM Tris–Cl) with protease inhibitor added (Roche complete mini-EDTA free). Pellets were passed through a 20-gauge needle and incubated on ice, then spun down. The supernatant was removed, and the pellet was resuspended in RIPA buffer with protease inhibitor added (Roche Complete). Pellets were again passed through a 27-gauge needle, incubated on ice and subsequently spun down. The supernatant was collected, and then incubated overnight at 4 °C on a rocker with either 12 µg of the anti-CUX1 antibody (B-10, Santa Cruz) or a rabbit IgG antibody. Protein A/G beads (Santa Cruz) were then added the following day to the supernatant, and incubated at 4 °C on a rocker for 1 h. The immunoprecipitated protein was then spun down, washed in cold PBS, resuspended in loading buffer, and subjected to SDS-PAGE.

### Sample preparation for LC–MS/MS

For the whole cell lysate samples, 20 μg of whole cell extract from KG-1 cells (determined by Bradford assay, Thermo #1856209 using λ595 nm) was loaded onto a 4–12% MOPS buffered 1D SDS-PAGE gel (Invitrogen NP0336BOX) and run at ~ 200 V for ~ 45 min. For the IP samples, 30% of the IP eluate (30 μL/100 μL) was loaded. The gel was stained with Imperial Stain (Thermo #24615) for 1 h at room temperature. For the whole cell lysate samples, the “p200” sections were excised from the gel by sterile razorblade (MW range 150–225 kDa) and the “p75” sections were excised in the MW range of ~ 60–75 kDa and in-gel trypsin digested as described below. For the IP samples, gel sections were excised as follows: “p200” sections MW range (~ 150–250 kDa) and “p75” sections MW range (~ 65–90 kDa) and in-gel trypsin digested as described below.

#### Trypsin digestion

Trypsin digestion was adapted from methods previously published^65,66^. In brief, gel sections were washed in dH_2_O and destained overnight using 100 mM NH_4_HCO_3_ (Sigma #285099) pH 7.5 in 50% acetonitrile (Fisher A998SK-4). A reduction step was performed by addition of 100 μL 50 mM NH_4_HCO_3_ pH 7.5 and 10 μL of 200 mM tris(2-carboxyethyl) phosphine HCl (Sigma #C4706-2G) at 37 °C for 30 min. The proteins were alkylated by addition of 100 μL of 50 mM iodoacetamide (Sigma #RPN6320V) prepared fresh in 50 mM NH_4_HCO_3_ pH 7.5 buffer and allowed to react in the dark at 20 °C for 30 min. Gel sections were washed in Millipore water, then acetonitrile, and vacuum dried. Trypsin digestion was carried out overnight at 37 °C with 1:50–1:100 enzyme–protein ratio of sequencing grade-modified trypsin (Promega #V5111) in 50 mM NH4HCO3 pH 7.5, and 20 mM CaCl2 (Sigma #C-1016). Peptides were extracted with 5% formic acid (Sigma #F0507-1L) in aqueous and 75% organic (ACN) combined and vacuum dried. Peptides were cleaned up using C18 spin columns (Thermo #89870) and sent to the Mayo Clinic Medical Genome Facility Proteomics Core for HPLC and LC–MS/MS data acquisition via Q-Exactive Orbitrap (Thermo).

### LC–MS/MS via MaxQuant

LC–MS/MS was performed using adapted methods previously published^67^. In brief, peptide samples were re-suspended in Burdick & Jackson HPLC-grade water containing 0.2% formic acid (Fluka #60-006-17), 0.1% TFA (Pierce #28903), and 0.002% Zwittergent 3–16 (Millipore Sigma #693023), a sulfobetaine detergent that contributes the following distinct peaks at the end of chromatograms: MH+ at 392, and in-source dimer [2 M+ H+] at 783, and some minor impurities of Zwittergent 3–12 seen as MH+ at 336. The peptide samples were loaded onto a 100 μm × 40 cm PicoFrit column self-packed with 2.7 μm Agilent Poroshell 120, EC-C18, washed, then switched in-line with a 0.33 uL Optimize EXP2 Stem Traps, packed spray tip nano column packed with Halo 2.7 μm Pep ES-C18, for a 2-step gradient. Mobile phase A was water/acetonitrile/formic acid (98/2/0.2) and mobile phase B was acetonitrile/isopropanol/water/formic acid (80/10/10/0.2). Using a flow rate of 350 nL/min, a 90 min, 2-step LC gradient was run from 5% B to 50% B in 60 min, followed by 50–95% B over the next 10 min, hold 10 min at 95% B, back to starting conditions and re-equilibrated.

Electrospray tandem mass spectrometry (LC–MS/MS) was performed at the Mayo Clinic Proteomics Core on a Thermo Q-Exactive Orbitrap mass spectrometer, using a 70,000 RP (70 K Resolving Power at 400 Da) survey scan in profile mode, *m/z* 340–1800 Da, with lockmasses, followed by 20 MSMS HCD fragmentation scans at 17,500 resolution on doubly and triply charged precursors. Single charged ions were excluded, and ions selected for MS/MS were placed on an exclusion list for 60 s. An inclusion list (generated with in-house software) consisting of expected Cux1 sequences was used during the LC–MS/MS runs.

### Database searching

Tandem mass spectra MS/MS samples were analyzed using MaxQuant (Max Planck Institute of Biochemistry, Martinsried, Germany; version 1.6.17.0). MaxQuant was set up to search the 210308_SPROT_Human_UP5640.fasta database assuming the digestion enzyme strict trypsin. MaxQuant was searched with a fragment ion mass tolerance of 20 PPM and a parent ion tolerance of 20 PPM. Maxquant 1FDR results files were processed in Perseus (version 1.6.14.0) for the proteingroups.txt in addition to being imported into Scaffold version 5.0.1.

### Criteria for protein identification

Scaffold (version Scaffold_5.0.1, Proteome Software Inc., Portland, OR) was used to validate MS/MS based peptide and protein identifications. Peptide identifications were accepted at 1% FDR by the Peptide Prophet algorithm (Keller et al. Anal. Chem. 2002;74(20):5383–5392) with Scaffold delta-mass correction. Protein identifications were accepted if they could be established at 1% FDR and contained at least 1 identified peptide. Protein probabilities were assigned by the Protein Prophet algorithm. Proteins sharing significant peptide evidence were grouped into clusters.

## Supplementary Information

Supplementary Figures.

Supplementary Table S1.

Supplementary Table S2.

Supplementary Table S3.

Supplementary Table S4.

## Data Availability

The mass spectrometry proteomic data sets (MK1) and (MK3) were uploaded to the ProteomeXchange consortium via the PRIDE partner repository with the dataset identifier PXD027527.
