# Supplementary material for: Genomic studies controvert the existence of the CUX1 p75 isoform
Source: Sci Rep. 2022 Jan 7;12:151. doi: 10.1038/s41598-021-03930-4 (PMC8741762; doi:10.1038/s41598-021-03930-4)
Supplement: Supplementary file 1 — Supplementary Figures. [file 41598_2021_3930_MOESM1_ESM.pdf]

Genomic studies controvert the existence of the CUX1 p75 isoform

Manisha Krishnan[1,3], Madhavi D. Senagolage[3], Jeremy T. Baeten[3], Donald J. Wolfgeher[2], Saira Khan[3], Stephen J. Kron[1,2,5] and Megan E. McNerney[1,3,4,5*]

Supplementary Information

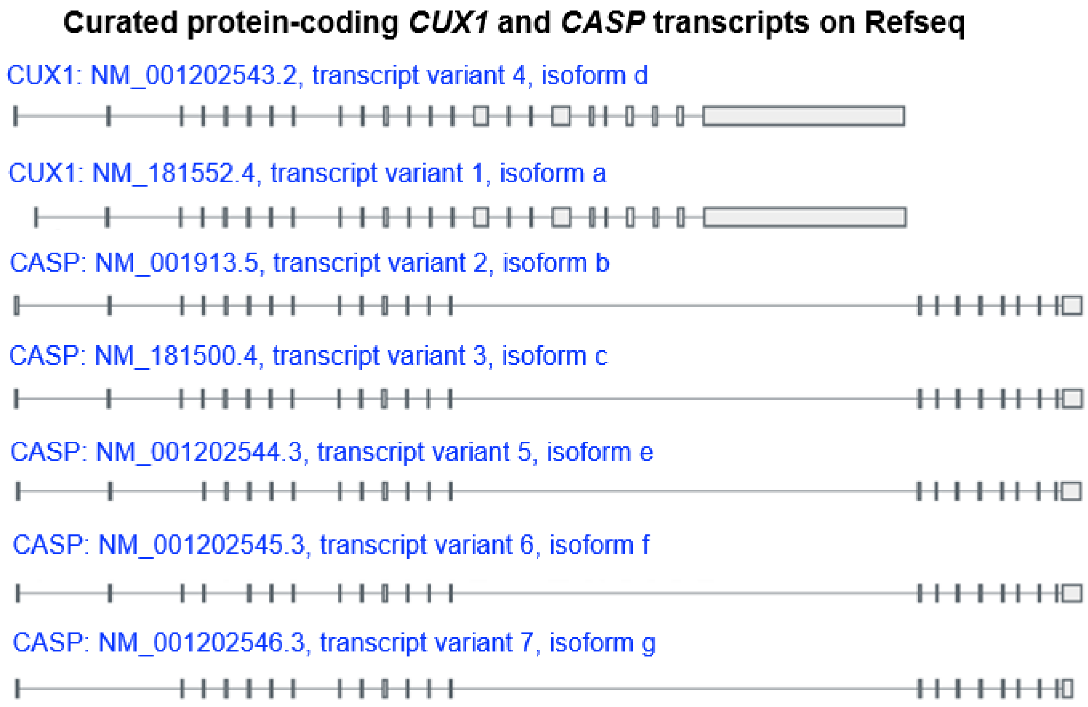

Supplementary Figure S1:

Schematic of seven predominant Refseq transcripts curated to be transcribed from the *CUX1* gene.

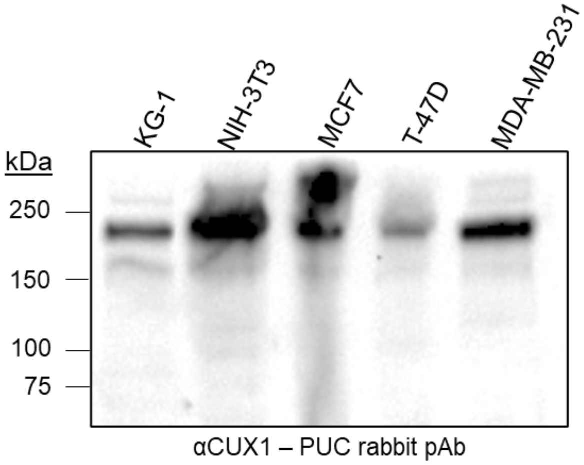

Supplementary Figure S2:

Immunoblot of CUX1 in the NIH-3T3 fibroblast line and several human breast cancer cell lines previously reported to express p75 CUX1 using the PUC antibody (n=3).

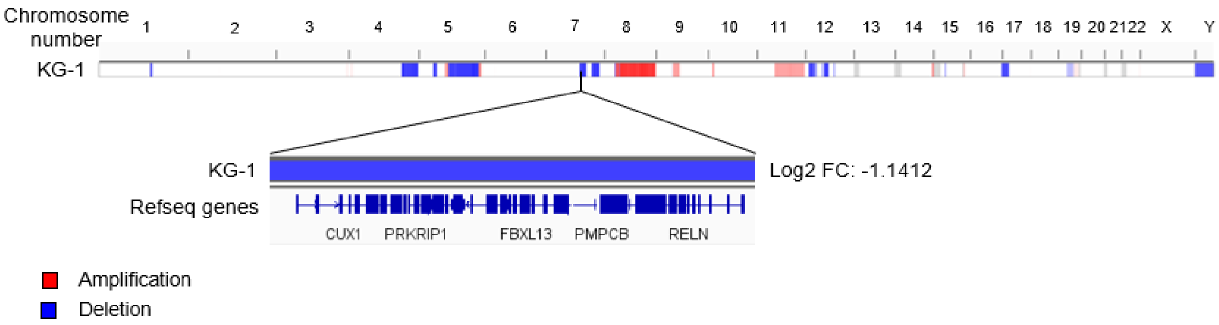

Supplementary Figure S3:

Copy number data for *CUX1* in the KG-1 cell line from the CCLE database (PMID: 22460905). Probe sets corresponding to a SNP allele or a copy number probe were used to infer copy number on the logarithmic scale, indicating a log2 fold change of -1.1412 for *CUX1* expression in the KG-1 cell line.

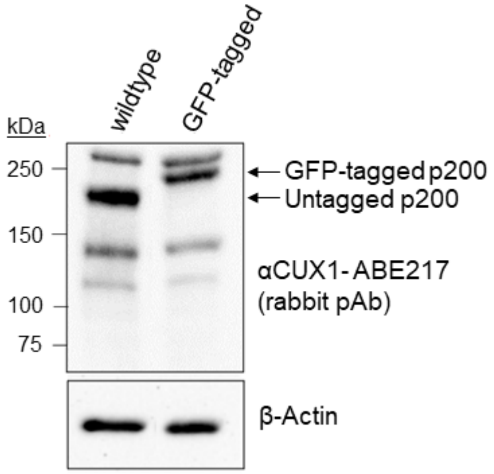

Supplementary Figure S4:

Immunoblot of CUX1 in a KG-1 cell line wherein endogenous CUX1 is tagged with GFP. Protein from wildtype KG-1 cells is also included. Blot has been cropped to remove an irrelevant intervening lane.

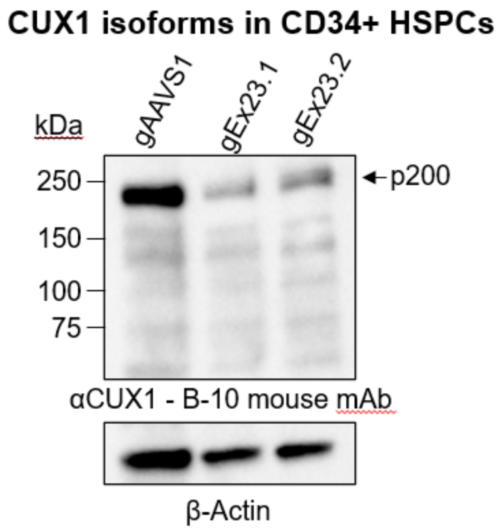

Supplementary Figure S5:

Immunoblot for CUX1 using the B-10 antibody in primary human CD34+ HSPCs. Bulk populations were edited with a control AAVS1 gRNA, or with one of two different gRNAs targeting exon 23 of *CUX1* (n=3).

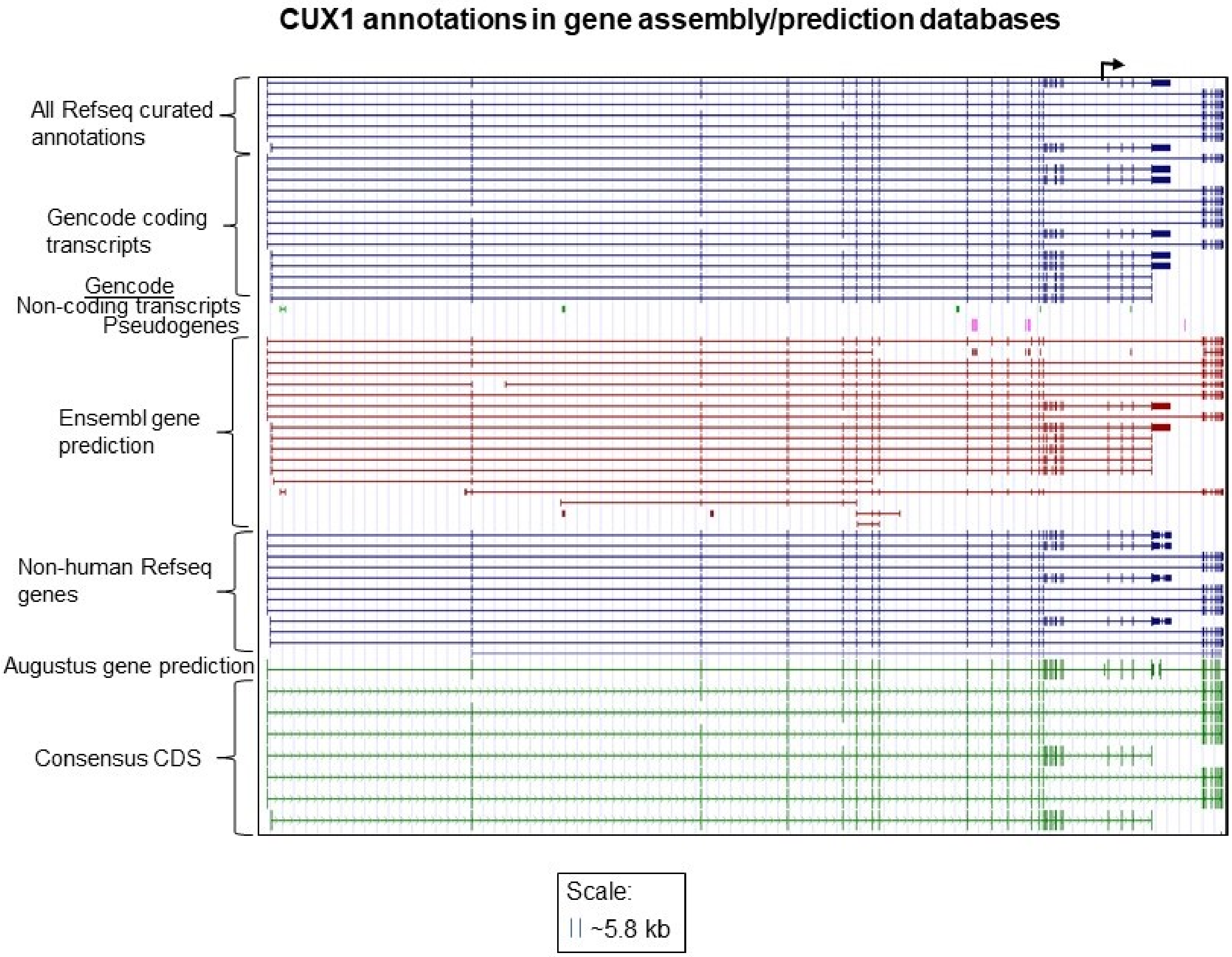

Supplementary Figure S6:

All CUX1 isoforms observed across several gene assembly and gene prediction databases (NCBI Refseq, Gencode, Ensembl, Augustus, CCDS) reveal no transcript resembling the p75 isoform. The arrow at the top of the figure indicates the putative intron 20 TSS for p75.

Uncropped images of blots and gels

Figure 1C

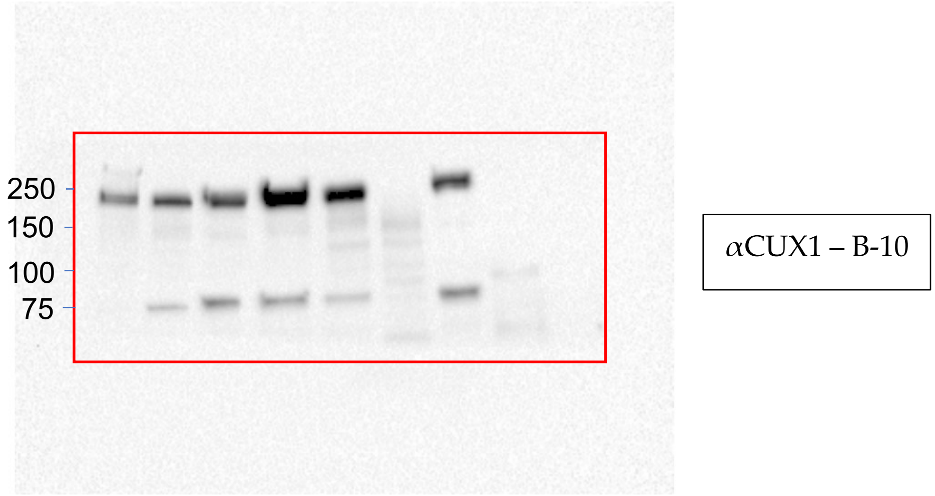

CUX1-B-10 blot in 8 human AML cell lines.

Figure 1D

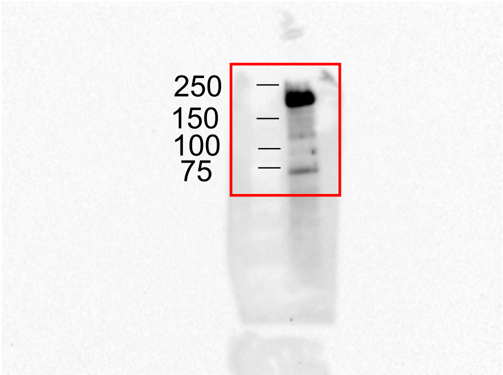

αCUX1 – B-10

CUX1-B-10 blot in primary human CD34+ HSPCs.

Figure 1E

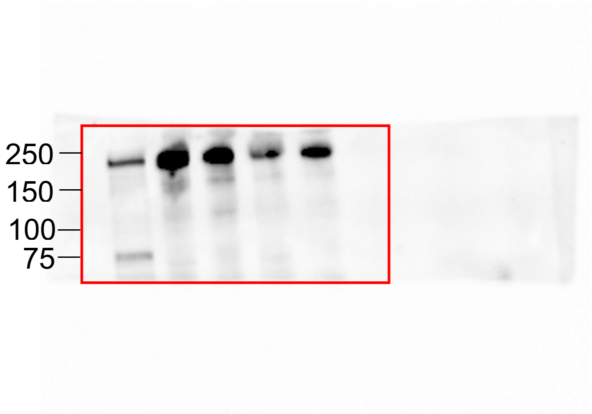

αCUX1 – B-10

CUX1 – B-10 blot in NIH-3T3 cells and human breast cancer cell lines previously reported to express p75.

Figure 1F

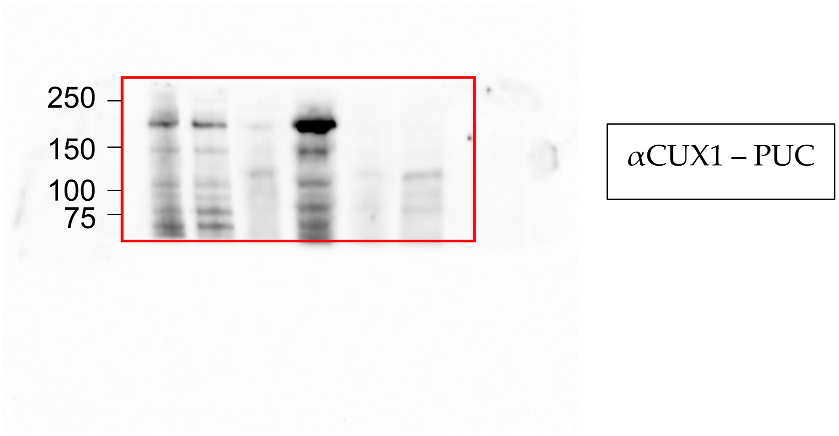

CUX1-PUC blot on 6 human AML cell lines.

Figure 1G

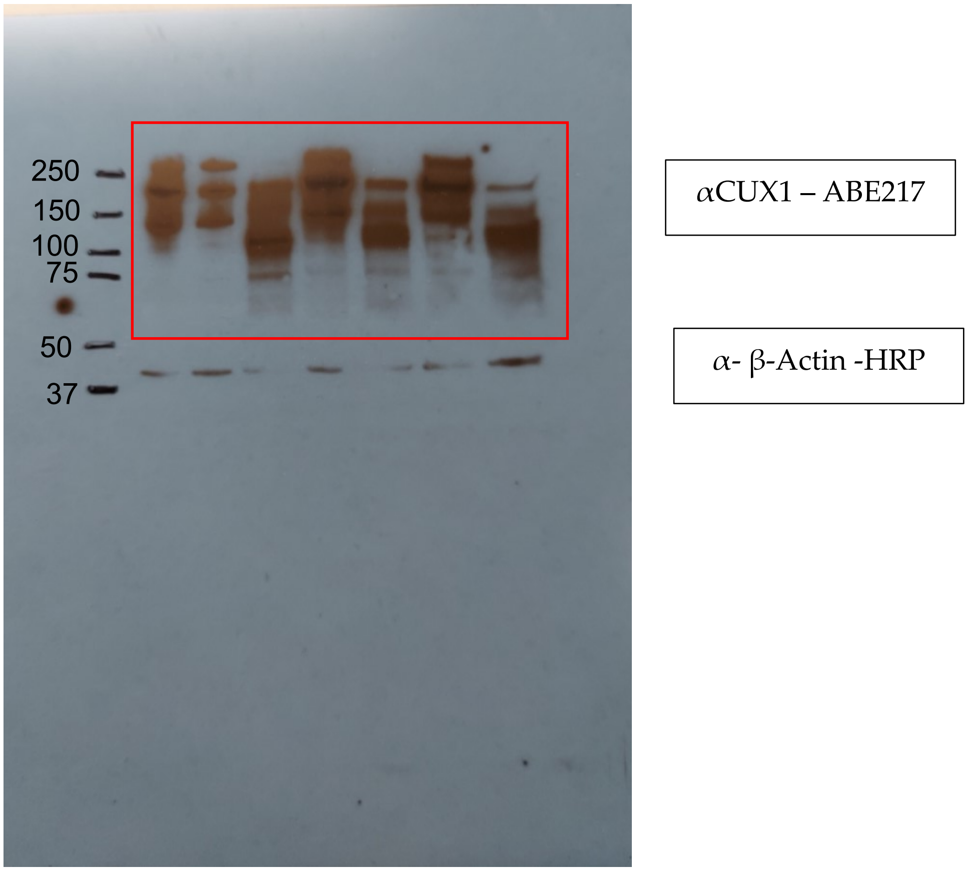

CUX1-ABE217 blot of 7 human AML cell lines (short exposure). β-actin loading control is also included below.

Figure 1H

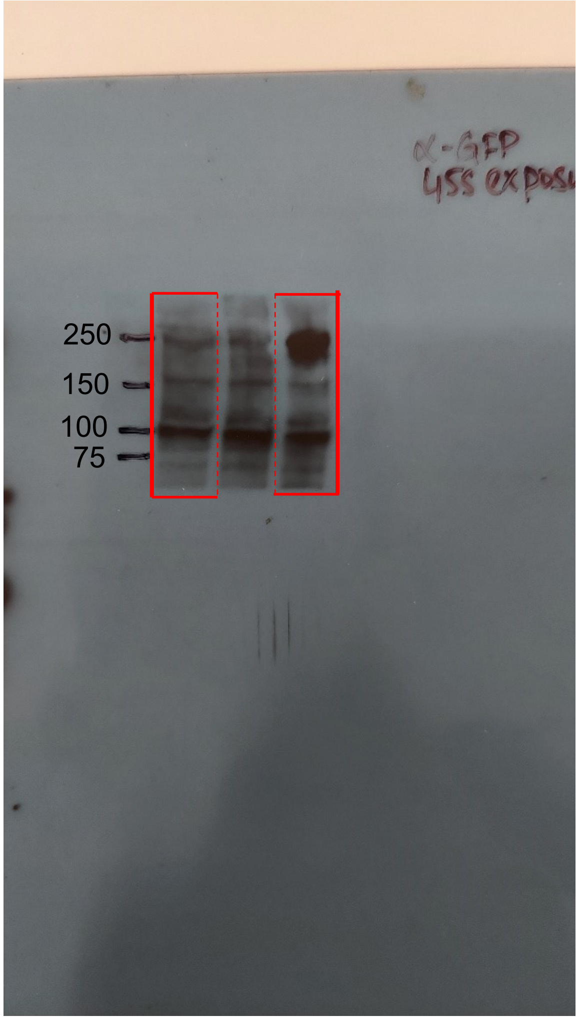

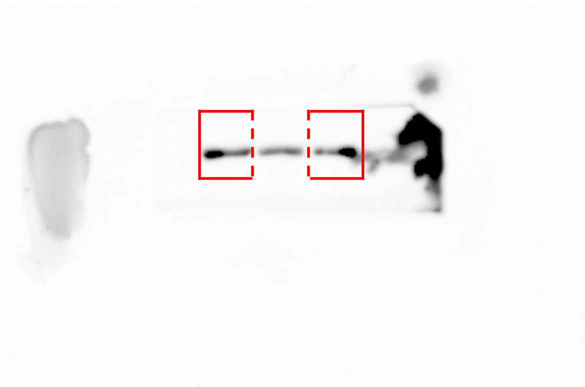

α- β-Actin -HRP

GFP blot of KG-1 cells where CUX1 is tagged with GFP on the left. β-actin loading control is shown on the right for the GFP blot. Membrane for the β-actin blot was cut from the same membrane for the GFP blot. Dotted lines show where the blot was cropped together to remove an irrelevant intervening lane.

Figure 2B

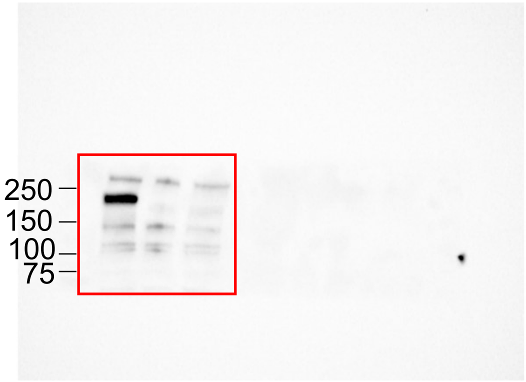

αCUX1 – ABE217

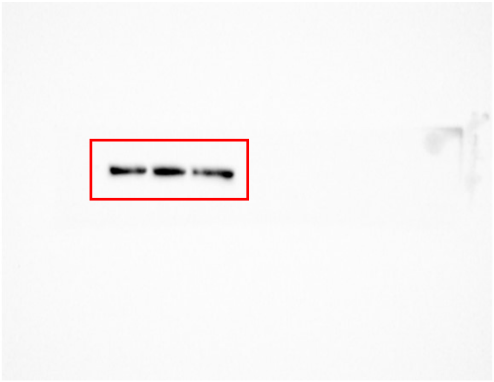

α- β-Actin -HRP

CUX1-ABE217 blot of KG-1 gEx4-edited single cell clones. β-actin loading control for the blot above is also included on the right. The membrane for the β-actin blot was cut from the same blot as the ABE217 blot.

Figure 2C

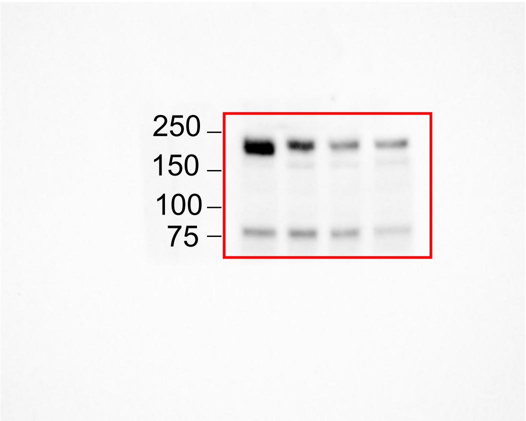

αCUX1 – B-10

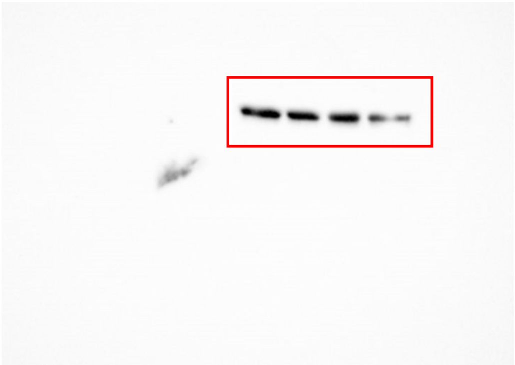

α- β-Actin -HRP

CUX1-B-10 blot of gEx4-edited KG-1 single-cell clones. β-actin loading control for Figure 2c is also shown on the right. Both blots were cut from the same membrane.

Figure 2E

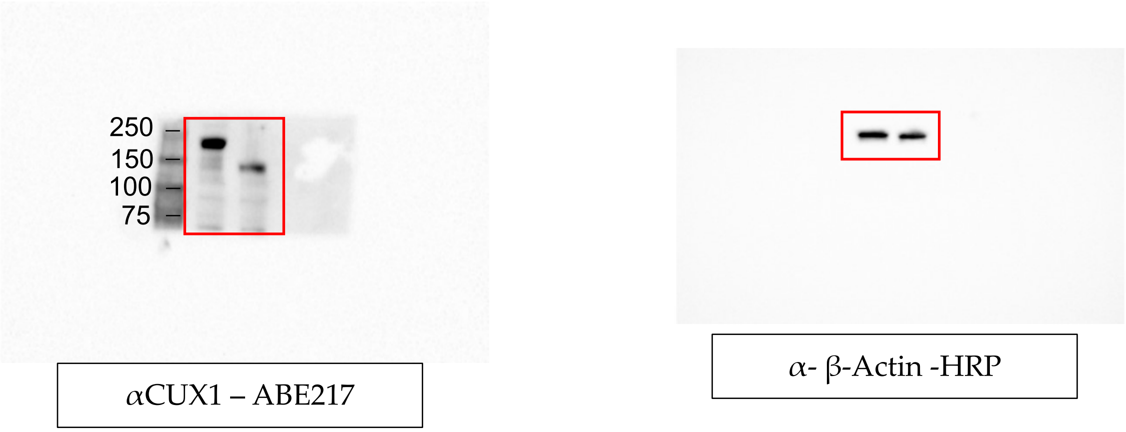

CUX1-ABE217 blot of gEx23-edited KG-1 clone. β-actin loading control for this blot is also shown on the right. Both blots were cut from the same membrane.

Figure 2F

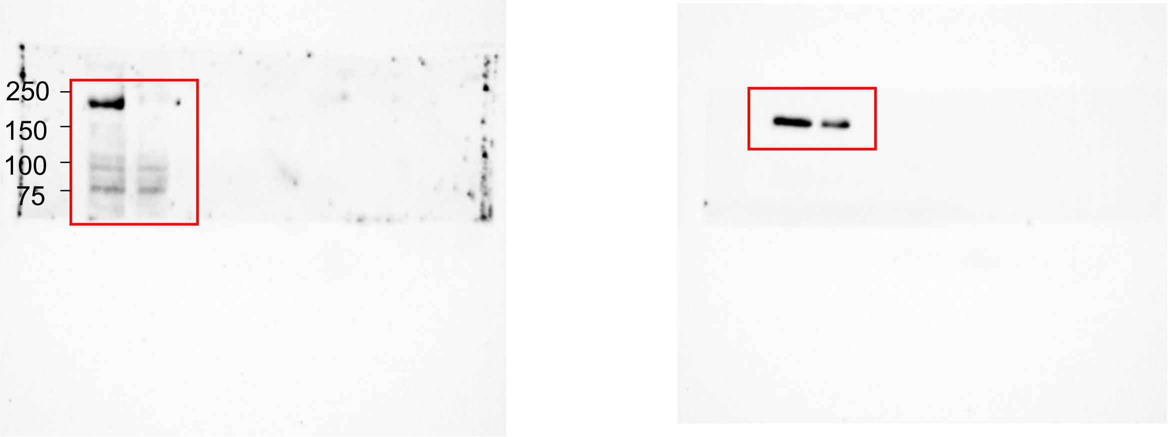

αCUX1 – B-10

α- β-Actin -HRP

CUX-B-10 blot of gEx23-edited KG-1 clone. β-actin loading control for this blot is also shown on the right. Both blots were cut from the same membrane.

Figure 3B

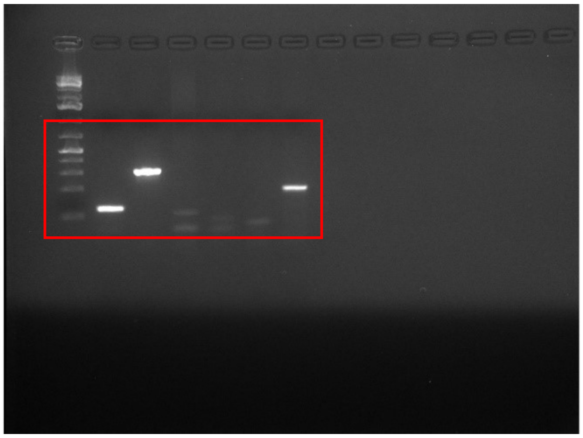

PCR products to identify clones with deletion of the p75 TSS using the intron 20 gRNAs.

Figure 3C

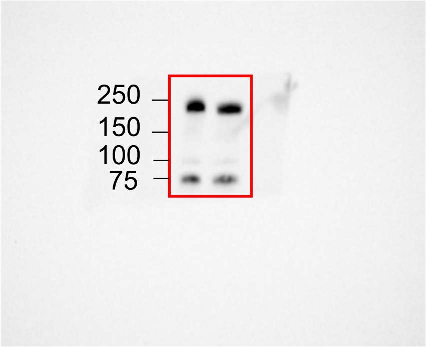

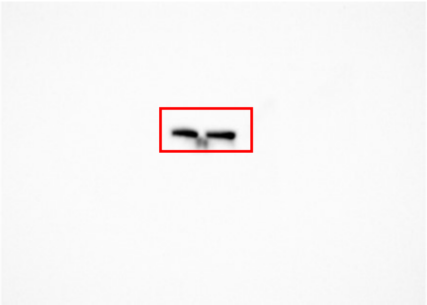

αCUX1 – B-10

α- β-Actin -HRP

CUX1-B-10 blot of a KG-1 clone with successful p75 TSS deletion. β-actin loading control for this blot is also shown. Blots were cut from the same membrane.

Figure 4A

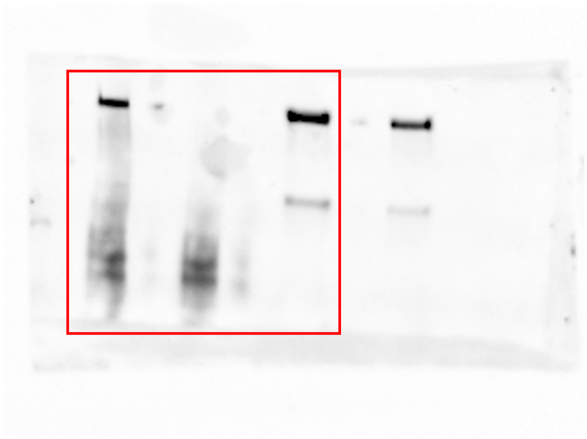

αCUX1 – B-10

CUX-1 western blot with B-10 after CUX1 immunoprecipitation with B-10 in the KG-1 cell line. Blot direction was reversed and blot was cropped for the final figure in 4A.

Figure 5C

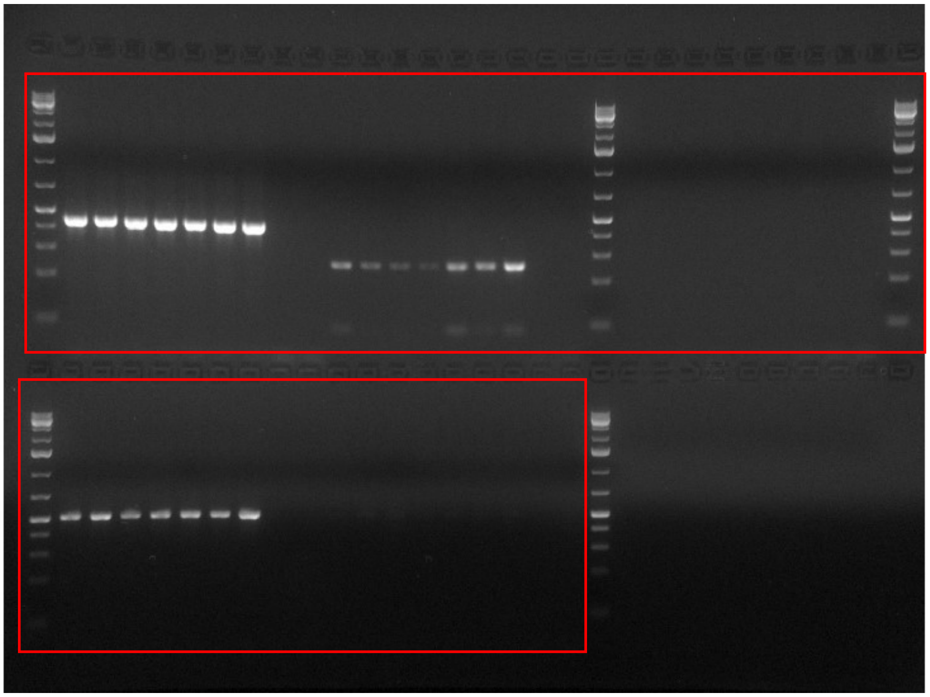

Reverse-transcriptase PCR products for GAPDH, p200 CUX1, and p75 CUX1 using different primer sets.

Supplementary Figure S2

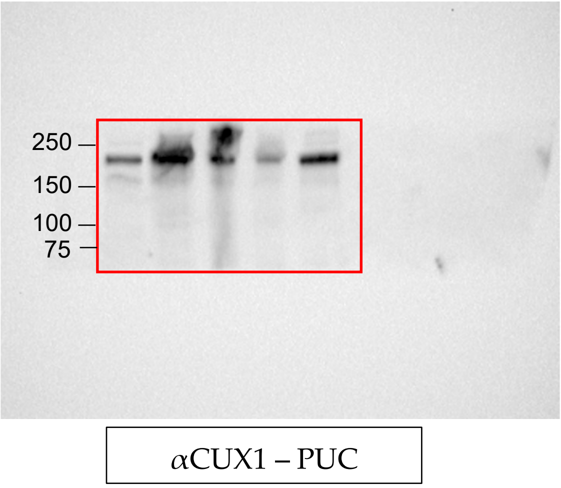

CUX1-PUC blot in NIH-3T3 cells and 3 human breast cancer cell lines.

Supplementary figure S4

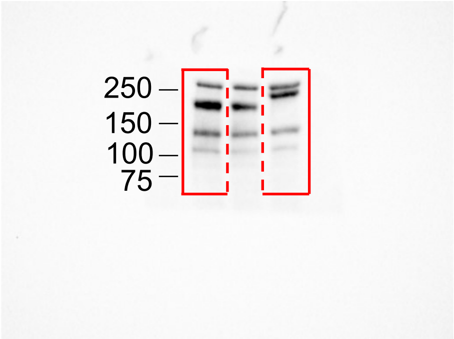

αCUX1 – ABE217

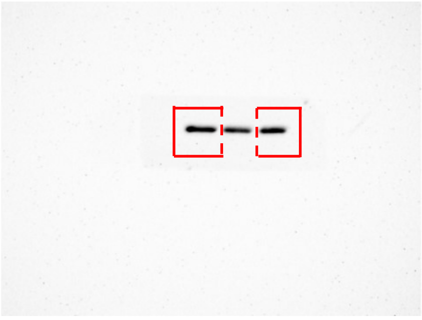

α- β-Actin -HRP

(Left) CUX1-ABE217 blot of CUX1-GFP tagged KG-1 cells. (Right) β-actin control of the CUX1-ABE217 blot, cut from the same membrane as the ABE217 blot. Red dotted lines on both blots indicate where the blot was cropped together to remove the irrelevant lane in the middle.

Supplementary Figure S5

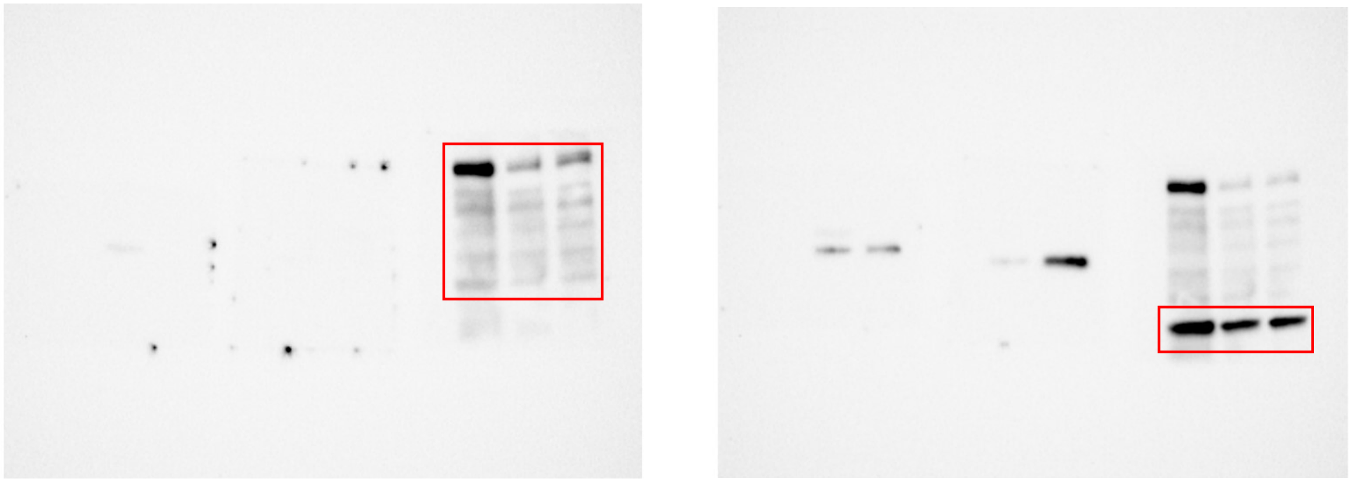

CUX1-B-10 blot for human CD34+ HSPCs edited with gEx23, with the β-actin loading control shown on the blot on the right.
